# Occupational Health Hazards: Employer, Employee, and Labour Union Concerns

**DOI:** 10.3390/ijerph18105423

**Published:** 2021-05-19

**Authors:** Oscar Rikhotso, Thabiso John Morodi, Daniel Masilu Masekameni

**Affiliations:** 1 Department of Environmental Health, Tshwane University of Technology, Private Bag X680, Pretoria 0001, South Africa; MorodiTJ@tut.ac.za; 2 Occupational Health Division, School of Public Health, University of Witwatersrand, Parktown 2193, South Africa; daniel.masekameni@wits.ac.za

**Keywords:** chemical and physical hazards, health hazard evaluation, health and safety standards, risk perception

## Abstract

This review paper examines the extent of employer, worker, and labour union concerns to occupational health hazard exposure, as a function of previously reported and investigated complaints. Consequently, an online literature search was conducted, encompassing publicly available reports resulting from investigations, regulatory inspection, and enforcement activities conducted by relevant government structures from South Africa, the United Kingdom, and the United States. Of the three countries’ government structures, the United States’ exposure investigative activities conducted by the National Institute for Occupational Safety and Health returned literature search results aligned to the study design, in the form of health hazard evaluation reports reposited on its online database. The main initiators of investigated exposure cases were employers, workers, and unions at 86% of the analysed health hazard evaluation reports conducted between 2000 and 2020. In the synthesised literature, concerns to exposure from chemical and physical hazards were substantiated by occupational hygiene measurement outcomes confirming excessive exposures above regulated health and safety standards in general. Recommendations to abate the confirmed excessive exposures were made in all cases, highlighting the scientific value of occupational hygiene measurements as a basis for exposure control, informing risk and hazard perception. Conclusively, all stakeholders at the workplace should have adequate risk perception to trigger abatement measures.

## 1. Introduction

Current occupational health and safety (OHS) legislation, in South Africa (SA), the United Kingdom (UK), and the United States (U.S.), makes provision for the reporting of complaints from exposure to occupational hazards by various stakeholders. The legislation was designed to ensure that working conditions encountered by workers employed in the various sectors is safe and healthy, as possible [1,2,3,4,5]. The reporting process is a function of national arrangement of the relevant OHS legislation from the respective countries. The overall enforcement regime of the OHS legislation, inclusive, has historically and continuously been criticised as being weak, overly bureaucratic, and dysfunctional [6,7,8].

Given that workers are in close proximity to occupational hazards, OHS legislation also places responsibility on them to report dangerous conditions to various stakeholders. This worker activism, provided for in the OHS legislation, can increase the effectiveness of legislation, which can translate to safety at work [6,8,9]. On the other hand, worker inaction in regards to reporting of dangerous conditions encountered at the workplace, can affect co-workers [6,10]. Accordingly, in recognition of this fact, OHS legislation worldwide places a duty on a worker to report dangerous conditions thereby protecting their own health and safety, as well as that of co-workers [1,2,4]. Given the longwinded and complicated bureaucracy associated with reporting procedures provided for in OHS legislation, workers are often deterred from reporting dangerous conditions with exposure remaining unabated [6,11]. A further deterrent to reporting dangerous conditions, following exhaustion of internal reporting procedures, is the weakness in the reporting regimes, asserts Spieler [6]. Undoubtedly, inadequate reporting of dangerous conditions encountered at work by various stakeholders is a contributory factor to the slow institution of regulatory interventions [6]. Complaints of exposure to occupational hazards is a crucial clue of how the various stakeholders perceive occupational hazards at the workplace. In the U.S., for example, Occupational Safety and Health Administration (OSHA) inspections are prioritised based on criteria that considers imminent danger situations, employee complaints, programmed inspections, and follow-up inspections [12]. However, employee complaints is reported to trigger expedited inspections [12,13]. To highlight the importance of worker activism in regard OHS, in a combined health risk assessment–occupational hazard appraisal conducted by New York City, occupational hazards identified by workers enabled the city to introduce intervention measures to mitigate exposure [14].

Worldwide, OHS legislation also provides for workers to refuse dangerous work [1,15,16]. This right of refusing dangerous work is however limited [17,18], as health and safety is a dual responsibility of the worker and employer [17]. Harcout and Harcout [17] also pointed out that current OHS legislation gives a right to management to command workers, further complicating the exercise of this right. This legal contradiction also leads to clashes between workers and management, often resulting is disciplinary action [17]. The right to refuse dangerous work is also burdensome to workers as they may be required to prove, through expert testimony or scientific evidence, of the existence of dangerous work [17,18]. This friction also leaves employees vulnerable to employer reprisals, argues Drapin [19].

The current review paper focused on the micro analysis of reports, where available, indicating stakeholder concerns in regard exposure to occupational health hazards. Lack of concern and inadequate risk perception by affected stakeholders in the management of identified occupational health hazards can result in adverse health impacts, especially on workers. In the U.S., the National Institute for Occupational Safety and Health (NIOSH) investigates workplace health hazards as well as offers technical and consultative assistance to various stakeholders through the discharge of legal authority given under the Occupational Safety and Health Act of 1970 (Section 20(a)(6)) [1], Code of Federal Regulation 1960.35(a)–(b) [20] and Code of Federal Regulations, title 42 volume 1 (section 85.1–85.12) [21]. In the UK, the Health and Safety at Work Act 1974 assigned similar functions to the Health and Safety Executive (HSE) [3]. In SA, the function of enforcing OHS legislation for general industry is mainly through the Department of Employment and Labour, as well as the National Department of Health to a lesser extent. The National Health Laboratory Service (which reports to the National Department of Health), through its subsidiary the National Institute for Occupational Health, serves as a referral body for occupational health matters [22].

This aforementioned scholarly review paper, part of a postgraduate study with the Tshwane University of Technology; ethical clearance: FCRE 2020/10/015 (FCPS 02) (SCI), investigates stakeholder concerns to occupational health hazard exposure as a function of reported and investigated exposure concerns.

## 2. Materials and Methods

### 2.1. Conceptual Framework

**Figure 1:**
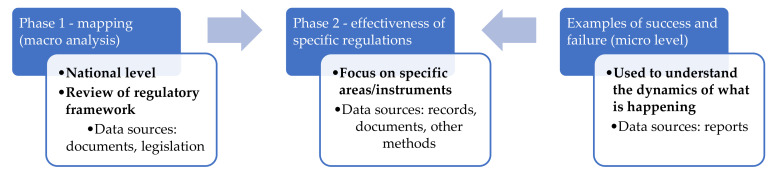
Conceptual framework.

The management of occupational health hazards follows from a hazard identification and risk assessment [23]. How the identified hazards and risks are treated thereafter is largely influenced by risk perception of different stakeholders inclusive of employers, workers and unions. Additionally, a country’s regulatory framework also plays an important role in hazard and risk perception. The micro analysis of available literature including reports issued by both labour inspectorates and supplementary institutions becomes necessary in order to gain insight on the subject matter. The conceptual framework, adapted from Hongoro and Kumaranayake [24], employed for this review study is shown in Figure 1.

### 2.2. Search Strategy

#### 2.2.1. Initial Search

An online search on databases of regulatory inspectorates from SA (Department of Employment and Labour), UK (HSE), and the U.S. (OSHA), recording stakeholder concerns or investigation requests to occupational health hazards was conducted as a first phase of the literature search. However, information in these inspectorates’ online databases including annual reports detailing conducted inspections were generic, complex (in the case of OSHA), and scant on the details set out as important search criteria adopted in this study. Thusly, these databases were excluded in the final adopted search strategy.

#### 2.2.2. Adopted Final Database Search and Search Strategy

The second phase of the literature search focused on databases of supplementary and legal bodies to the inspectorates. Consequently, the final adopted database search only focused on the National Institute of Occupational Safety and Health (NIOSH) Health Hazard Evaluations (HHEs) reports as they yielded results matching the adopted search criteria for this review paper. The HHE report database provides a repository of all completed NIOSH-led workplace investigations, a topic of concern for this current study. As of March 2021, the repository had 3614 HHE reports dating back to 1972, covering all sectors. This study however targeted HHEs conducted over two decades spanning from the year 2000 to 2020, conducted within the manufacturing sector. As per the database repository structure, the literature search was refined to include “All States/OSHA Regions”, “Manufacturing”, “all industry subcategories”, and “all health effects”.

### 2.3. Inclusion Criteria and Exclusion Criteria

**Figure 2:**
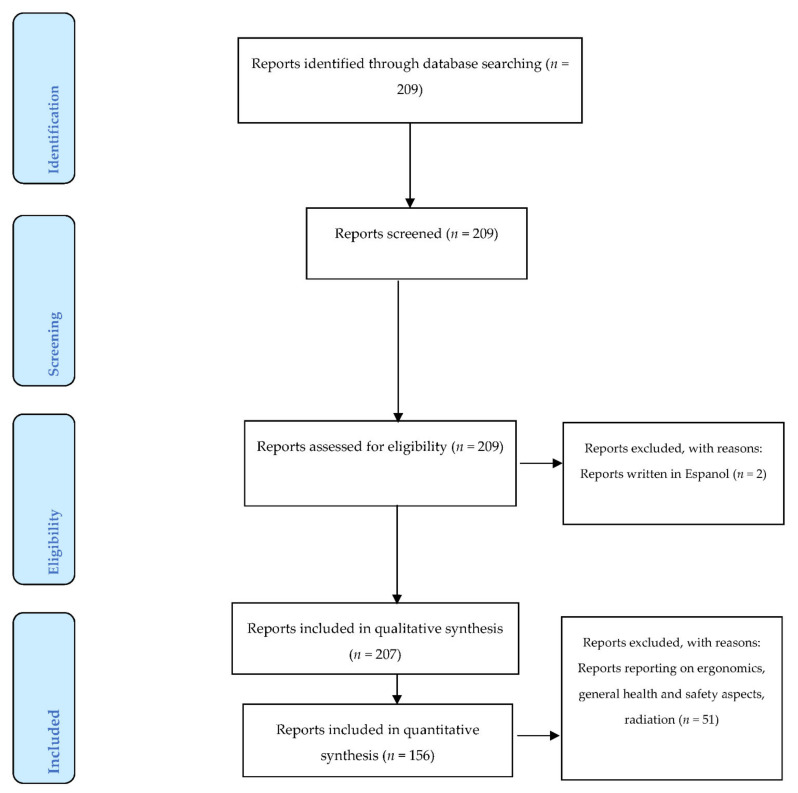
PRISMA flow diagram.

Reports considered in the final synthesis were those published in English. Other criteria used in the inclusion and exclusion criteria are shown in Figure 2. Between 2000 and 2020, a total of 209 HHE reports were reposited in the database. Of this total, only two HHE reports were excluded in the final analysis as they were published in Spanish. The qualitative analysis included a total 207 HHE reports whilst the quantitative analysis only included 155 reports. The qualitative analysis focused and reports on industry type and initiator(s) of the investigations on the one hand. On the other hand, the quantitative analysis focused and reports on the target occupational health stressor, type of sample (mainly personal and area samples, as appropriate), measured exposure levels and comparison of the exposure levels to health and safety standards prescribed and recommended by various countries and governmental agencies. The quantitative synthesis further excluded 51 HHE studies reporting on ergonomic, radiation, and hazardous biological agents, due to textual complexities associated with reporting and interpreting results derived therefrom.

## 3. Results

In general, the synthesised literature suggests that NIOSH is discharging its designated legal duties given under the Occupational Safety and Health Act 1970 [1], Code of Federal Regulation 1960.35(a)–(b) [20], and Code of Federal Regulations, title 42 volume 1 (Section 85.1–85.12) [21]. The synthesised literature revealed that the manufacturing industry exposes workers to a myriad of chemical, physical, ergonomic, and biological occupational health hazard types, with varying degrees of exposure.

### 3.1. Qualitative Analysis

Table 1 provides an overview, qualitatively, of the initiators of the NIOSH-led exposure investigations included. Overall, employers at (*n* = 87(42%)); employees at (*n* = 59(28.5%)); and unions at (*n* = 32(15.5%)) were the chief initiators of workplace investigations for the period from 2000 to 2020, which when combined, contributed 86% of the investigations. The agency also offered technical and consultative assistance over the period to other government agencies or departments with investigated cases at (*n* = 10(4.9%)). Investigations conducted as part of state programmes at (*n* = 3(1.4%)) were also discharge during the period. Joint requests by different stakeholders were also recorded during the period. Workers are empowered by OHS legislation to request investigations following concerns of exposure to occupational health hazards [1,2,3,4,20,21], as is the case with the cases reported to NIOSH.

### 3.2. Quantitative Analysis

Table 2 shows the quantitative data used as input for making judgements relating to exposure for each investigated case. The quantitative exposure data in Table 2 was derived using occupational hygiene measurement techniques as part of the field investigations, and employed personal and area measurements, as appropriate. Concern of exposure to chemical hazard types were, by far, the most investigated compared to physical hazards. Undoubtedly, the manufacturing sector involves the handling of substances with resultant exposures to a myriad of chemical hazards [25,26], some of which are currently not regulated. Comparison of the measured air concentrations of these chemical hazards, both personal and area measurements, generally showed a mixed view in relation to measured exposure levels in compliance with (and non-compliance with) the assigned health and safety standards. In the absence of international covenants on health and safety standards, compliance to these standards becomes a function of the selected standard, in some instances. These occupational hygiene measurements provide objective evidence of the severity of the risks, wherefrom the need for instituting mitigating measures could be proposed [27]. The quantified exposures for both chemical and physical hazard types exceeding the health and safety standards justified stakeholder concerns and adjudged to be indicative of adequate hazard and risk perception on the part of the initiators.

Of the investigated and quantified chemical hazards, diacetyl levels from the popcorn manufacturing industry exceeded the NIOSH recommended exposure level in almost all cases. Whereas noise exposures above the regulated exposure limits were noted in almost all investigated and quantified physical hazards. The measured noise levels exceeded both the NIOSH recommended exposure level as well as the OSHA permissible exposure level. Additionally, exposure to heat stress was also prevalent in the included investigations.

The workplaces at which these investigations were conducted were enabled to abate hazards, in most instances, highlighting the positive impact of these investigative activities. Actions taken to abate identified hazards serve as evidentiary proof of employers fulfilling their legal responsibility of providing safe and healthy workplaces.

**Table 1:** Qualitative overview of initiators of NIOSH–HHE investigations included.

	Initiator						
Industry Type, Year (Reference)	Employer	Employee(s)	State Program	Union	Government Agency	Technical Assistance	Other
Coffee roasting, flavouring, and packaging facility, 2020 [28]	-	✓	-	-	-	-	-
Coffee roasting, flavouring, and packaging facility, 2020 [29]	✓	-	-	-	-	-	-
Architectural metal fabrication workshop, 2020 [30]	✓	-	-	-	-	-	-
Coffee roasting and packaging facility, 2019 [31]	✓	-	-	-	-	-	-
Electronics recycling company, 2019 [32]	✓	-	-	-	-	-	-
Coffee roasting and packaging facility and two off-site retail cafes, 2019 [33]	✓	-	-	-	-	-	-
Rubber manufacturing facility, 2019 [34]	-	✓	-	-	-	-	-
Paper converting equipment manufacturing facility, 2019 [35]	-	✓	-	-	-	-	-
Coffee roasting, flavouring, and packaging facility, 2019 [36]	✓	-	-	-	-	-	-
Brewery, 2019 [37]	-	-	-	✓	-	-	-
Aircraft power plant parts manufacturer, 2019 [38]	-	✓	-	-	-	-	-
Precast concrete manufacturer, 2019 [39]	✓	-	-	-	-	-	-
Automobile manufacturer, 2019 [40]	-	✓	-	-	-	-	-
Ceramic tile manufacturer, 2019 [41]	✓	-	-	-	-	-	-
Optical media production company, 2018 [42]	✓	-	-	-	-	-	-
Coffee roasting and packaging facility, 2018 [43]	✓	-	-	-	-	-	-
Coffee roasting and packaging facility, 2018 [44]	✓	-	-	-	-	-	-
Coffee roasting and packaging facility, 2018 [45]	✓	-	-	-	-	-	-
Steel coil pickling plant, 2018 [46]	✓	-	-	-	-	-	-
Fiberglass insulation manufacturing plant; and residential clothes dryers manufacturing, 2018 [47]	✓	-	-	-	-	-	-
Flooring manufacturing plant, 2018 [48]	-	✓	-	-	-	-	-
Bullet manufacturer, 2018 [49]	✓	-	-	-	-	-	-
Engine machining plant, 2018 [50]	-	-	-	✓	-	-	-
Battery manufacturing plant, 2018 [51]	✓	-	-	-	-	-	-
Pet care product manufacturing, 2018 [52]	✓	-	-	-	-	-	-
Coffee roasting and packaging facility, 2018 [53]	✓	-	-	-	-	-	-
Coffee roasting and packaging facility, 2018 [54]	✓	-	-	-	-	-	-
Two coffee roasting and packaging facility, 2018 [55]	✓	-	-	-	-	-	-
Coffee roasting and packaging facility, 2018 [56]	-	✓	-	-	-	-	-
Coffee roasting and packaging facility, 2018 [57]	✓	-	-	-	-	-	-
Coffee roasting and packaging facility, 2018 [58]	✓	-	-	-	-	-	-
3-D printing product manufacturing facility, 2017 [59]	✓	-	-	-	-	-	-
Aircraft equipment depot, 2017 [60]	✓	-	-	-	-	-	-
Plastic film assembly facility, 2017 [61]	✓	-	-	-	-	-	-
Water heater manufacturing, 2017 [62]	-	-	-	✓	-	-	-
Coffee processing facility, 2017 [63]	✓	-	-	-	-	-	-
Coffee roasting and packaging facility and attached retail café, 2017 [64]	✓	-	-	-	-	-	-
Coffee processing plant, 2017 [65]	✓	-	-	-	-	-	-
Grey and ductile iron foundry, 2017 [66]	✓	-	-	-	-	-	-
Coffee roasting and packaging facility, 2017 [67]	✓	-	-	-	-	-	-
Coffee roasting and packaging facility, 2017 [68]	✓	-	-	-	-	-	-
Poultry production plant **, 2017 [69]	✓	-	-	-	✓	-	✓
Poultry production plant **, 2016 [70]	✓	-	-	-	✓	-	✓
Stone countertop manufacturing plant, 2016 [71]	-	-	-	-	✓	-	-
Hammer forge company, 2016 [72]	-	-	-	✓	-	-	-
Riffle barrel manufacturing, 2016 [73]	✓	-	-	-	-	-	-
Security portal manufacturer, 2016 [74]	✓	-	-	-	-	-	-
Automobile parts manufacturing plant, 2016 [75]	✓	-	-	-	-	-	-
Steel building materials manufacturer, 2016 [76]	✓	-	-	-	-	-	-
Snack foods manufacturing facility, 2016 [77]	-	✓	-	-	-	-	-
Coal and copper slag processing facility, 2016 [78]	✓	-	-	-	-	-	-
Syntactic foam manufacturing facility, 2016 [79]	-	✓	-	-	-	-	-
Fiberglass-reinforced wind turbine blade manufacturing, 2016 [80]	✓	-	-	-	-	-	-
Automotive engine water pump manufacturer, 2016 [81]	✓	-	-	-	-	-	-
Garlic paste production process, 2015 [82]	✓	-	-	-	-	-	-
Aircraft ejection seat manufacturer, 2015 [83]	✓	-	-	-	-	-	-
Poultry processing plant, 2015, [84]	✓	-	-	-	-	-	-
Grey and ductile iron foundry 2015 [85]	✓	-	-	-	-	-	-
Dry cleaning shop, 2015 [86]	✓	-	-	-	-	-	-
Orthopaedic implant manufacturer, 2015 [87]	-	✓	-	-	-	-	-
Label manufacturing facility, 2014 [88]	✓	-	-	-	-	-	-
Polymer additive manufacturing facility, 2014 [89]	-	✓	-	-	-	-	-
Aircraft engine services facility, 2014 [90]	-	✓	-	-	-	-	-
Specialty Chemicals plant, 2014 [91]	-	✓	-	-	-	-	-
Pet food manufacturing facility, 2014 [92]	-	✓	-	-	-	-	-
Electrical cables accessories manufacturing, 2014 [93]	-	✓	-	-	-	-	-
Poultry processing plant, 2014 [94]	-	-	-	-	✓	-	-
Automotive lead-acid battery recycling company, 2014 [95]	-	✓	-	-	-	-	-
Steel mill fiberglass fibre shedding **, 2013 [96]	-	✓	-	✓	-	-	✓
Furniture manufacturing plant, 2013 [97]	✓	-	-	-	-	-	-
Poultry processing plant *, 2013 [98]	-	-	-	-	✓	✓	✓
Cream cheese manufacturing facility, 2013 [99]	-	✓	-	-	-	-	-
Snack food production facility, 2013 [100]	-	✓	-	-	-	-	-
Flavouring manufacturing facility, 2013 [101]	-	✓	-	-	-	-	-
Poultry breading plant, 2013 [102]	-	-	-	✓	-	-	-
Tire manufacturing plant, 2013 [103]	-	✓	-	-	-	-	-
Aluminium beverage can manufacturing, 2012 [104]	-	✓	-	-	-	-	-
Poultry processing plant, 2012 [105]	✓	-	-	-	-	-	-
Eyeglass manufacturing, 2012 [106]	✓	-	-	-	-	-	-
Abrasive blasting, 2012 [107]	✓	-	-	-	-	-	-
Poultry processing facility, 2012 [108]	✓	-	-	-	-	-	-
Indium-tin oxide production facility, 2012 [109]	✓	-	-	-	-	-	-
Aircraft engine manufacturing facility, 2012 [110]	-	-	-	✓	-	-	-
Brewery, 2011 [111]	-	-	-	✓	-	-	-
Drum refurbishing plant, 2011 [112]	-	✓	-	-	-	-	-
Ink ribbon manufacturing, 2011 [113]	✓	-	-	-	-	-	-
Aluminium smelter, 2011 [114]	-	✓	-	-	-	-	-
Flavouring manufacturing company, 2011 [115]	-	-	-	✓	-	-	-
Semiconductor manufacturing plant, 2011 [116]	✓	-	-	-	-	-	-
Immortalis Botanicals, 2010 [117]	✓	-	-	-	-	-	-
Steel manufacturing, 2010 [118]	-	✓	-	-	-	-	-
Workholding manufacturing facility, 2010 [119]	✓	-	-	-	-	-	-
Electrolytic manganese dioxide processing plant [120]	-	-	-	✓	-	-	-
Aircraft manufacturing plant, 2010 [121]	-	✓	-	-	-	-	-
Steel grating manufacturing plant **, 2009 [122]	✓	-	-	✓	-	-	✓
Road markings manufacturing, 2009 [123]	-	✓	-	-	-	-	-
Road sign printing, 2009 [124]	✓	-	-	-	-	-	-
Metal furniture manufacturing, 2009 [125]	-	✓	-	-	-	-	-
Printed circuit board manufacturing, 2009 [126]	-	✓	-	-	-	-	-
Bakery, 2009 [127]	-	✓	-	-	-	-	-
Flavourings, modified dairy products, and bacterial additive manufacturing, 2009 [128]	-	✓	-	-	-	-	-
Tungsten carbide manufacturing, 2009 [129]	-	✓	-	-	-	-	-
Three commercial kitchens, 2009 [130]	-	-	-	✓	-	-	-
Automotive parts manufacturing, 2008 [131]	-	-	-	✓	-	-	-
Turkey processing plant, 2008 [132]	-	-	-	✓	-	-	-
Cabinet mill and assembly plant, 2008 [133]	✓	-	-	-	-	-	-
Piston and cylinder liner manufacturing plant, 2008 [134]	-	-	-	✓	-	-	-
Automotive parts manufacturing, 2008 [135]	-	-	-	✓	-	-	-
Pottery shop, 2008 [136]	✓	-	-	-	-	-	-
Entek Manufacturing *, 2008 [137]	-	-	-	-	✓	✓	✓
Metal conduit manufacturing, 2008 [138]	-	✓	-	-	-	-	-
Flavouring manufacturing plant, 2008 [139]	-	-	✓	-	-	-	-
Glass bottle manufacturing, 2007 [140]	-	✓	-	-	-	-	-
Liquid and powdered flavouring manufacturer, 2007 [141]	-	-	✓	-	-	-	-
Roller chain manufacturing facility, 2007 [142]	-	-	-	✓	-	-	-
Smelter, 2007 [143]	-	-	-	✓	-	-	-
Specialty steel manufacturing, 2007 [144]	-	-	-	✓	-	-	-
Communications company, 2007 [145]	-	✓	-	-	-	-	-
Poultry processing facility, 2007 [146]	-	-	-	-	-	✓	-
Popcorn popping plant, 2007 [147]	✓	-	-	-	-	-	-
Label distribution company, 2007 [148]	✓	-	-	-	-	-	-
Flavouring manufacturing plant *, 2007 [149]	-	-	-	-	✓	✓	✓
Ballistic systems manufacturing, 2006 [150]	✓	-	-	-		-	-
Tapered steel roller bearing manufacturing, 2006 [151]	-	-	-	✓	-	-	-
Motorcycle assembly facility, 2006 [152]	-	✓	-	-	-	-	-
Microwave popcorn plant, 2006 [153]	-	-	-	-	-	✓	-
Polystyrene and foam manufacturing, 2006 [154]	-	✓	-	-	-	-	-
Flock manufacturing facility, 2006 [155]	-	-	-	-	-	✓	-
Automotive assembly plant, 2006 [156]	-	✓	-	-	-	-	-
Cultured marble vanities, bath tubs, and shower walls and floors manufacturing, 2006 [157]	-	✓	-	-	-	-	-
Aircraft fuel cells manufacturing, 2006 [158]	-	✓	-	-	-	-	-
Poultry processing facility, 2006 [159]	✓	-	-	-	-	-	-
Residential and industrial furnace manufacturing, 2006 [160]	-	-	-	✓	-	-	-
Glass container manufacturer, 2005 [161]	-	-	-	✓	-	-	-
Computer services, 2005 [162]	-	✓	-	-	-	-	-
Fabricated metal product manufacturing, 2005 [163]	-	✓	-	-	-	-	-
PTFE, thermoplastic rotating seals, subassembly systems and plastic mating component manufacturing, 2005 [164]	-	✓	-	-	-	-	-
Portland cement company, 2005 [165]	✓	-	-	-	-	-	-
Ice cream and frozen novelty product manufacturer, 2005 [166]	-	-	-	✓	-	-	-
Hardware (zinc casting department), 2005 [167]	-	✓	-	-	-	-	-
Axle assembly facility, 2005 [168]	-	✓	-	-	-	-	-
Magnesium ingot, magnesium recycling and chemical by-products supplier and manufacturer *, 2005 [169]	✓	-	-	✓	✓	-	✓
Asphalt plant 1, 2005 [170]	-	✓	-	-	-	-	-
Heavy metal fabrication operation, 2005 [171]	✓	-	-	-	-	-	-
Corrugated cardboard and pulp paper production facility, 2004 [172]	-	-	-	-	-	✓	-
Microwave popcorn plant *, 2004 [173]	-	-	-	-	✓	✓	✓
Microwave popcorn production, 2004 [174]	-	✓	-	-	-	-	-
Corrosive-resistant stainless steel and piping system fabrication facility, 2004 [175]	-	-	-	✓	-	-	-
Metal parts manufacturing, 2004 [176]	✓	-	-	-	-	-	-
Polyethylene and polypropylene plastics complex, 2004 [177]	-	-	-	✓	-	-	-
Coal-fired boiler component fabrication, 2004 [178]	✓	-	-	-	-	-	-
Milk, ice cream and cultured dairy products processor, 2004 [179]	-	✓	-	-	-	-	-
Agri-business enterprise (potato processor), 2004 [180]	✓	-	-	-	-	-	-
Wireless network systems manufacturer, 2004 [181]	-	✓	-	-	-	-	-
Microwave popcorn plant, 2003 [182]	✓	-	-	-	-	-	-
Foam cushion manufacturer, 2003 [183]	-	-	-	-	✓	-	-
Specialty chemical manufacturer, 2003 [184]	-	-	-	-	-	✓	-
Custom concrete counter tops manufacturer, 2003 [185]	✓	-	-	-	-	-	-
Aluminium oil cooler producer, 2003 [186]	✓	-	-	-	-	-	-
Turkey processing facility, 2003 [187]	-	-	-		-	✓	-
Flexographic printing operation, 2003 [188]	✓	-		-	-	-	-
Microwave popcorn plant, 2003 [189]	-	✓	-	-	-	-	-
Metal valves and steam traps manufacturer, 2003 [190]	✓	-	-	-	-	-	-
Metal phosphide-based fumigant manufacturer, 2003 [191]	-	✓	-	-	-	-	-
Flexible packaging and pressure sensitive material manufacturer, 2003 [192]	-	-	-	✓	-	-	-
Advanced surgical instruments and medical services developer, 2003 [193]	✓	-	-	-	-	-	-
Microwave popcorn manufacturer, 2003 [194]	-	-	✓	-	-	-	-
Valve manufacturing, 2002 [195]	✓	-	-	-	-	-	-
Titanium and aluminium commercial airplane parts manufacturer, 2002 [196]	-	✓	-	-	-	-	-
Electroplated strip steel manufacturer, 2002 [197]	-	-	-	✓	-	-	-
Rubber moulded parts, rubber to metal mould bonded bushings, Teflon lined bonded bushings, and rubber compounds manufacturer, 2002 [198]	-	-	-	✓	-	-	-
Air compressor manufacturer, 2002 [199]	✓	-	-	-	-	-	-
Sofa cushion manufacturer, 2002 [200]	-	✓	-	-	-	-	-
Neon tube manufacturing, 2002 [201]	✓	-	-	-	-	-	-
Flexographic printing operation, 2002 [202]	✓	-	-	-	-	-	-
Glass funnel and panel manufacturer, 2002 [203]	-	-	-	✓	-	-	-
Automotive brake calipers and drum manufacturer **, 2002 [204]	✓	-	-	✓	-	-	✓
Automatic transmissions and transmission components manufacturer **, 2002 [205]	✓	✓	-	✓	-	-	✓
Seat cushion manufacturer, 2002 [206]	-	-	-	-	✓	-	-
Specialty, nonferrous metal-alloy billet producer, 2001 [207]	-	✓	-	-	-	-	-
Potato product manufacturer, 2001 [208]	-	✓	-	-	-	-	-
Catalyst manufacturer, 2001 [209]	-	-	-	✓	-	-	-
Wire rope products manufacturer, 2001 [210]	-	✓	-	-	-	-	-
Instrumentation and component manufacturer, 2001 [211]	-	✓	-	-	-	-	-
Woodworking operation (garage interior component production), 2001 [212]	-	✓	-	-	-	-	-
Shear, scissors and thread manufacturer, 2001 [213]	✓	-	-	-	-	-	-
Nonwoven and specialty fibres manufacturer, 2001 [214]	-	✓	-	-	-	-	-
Portland cement company, 2001 [215]	-	-	-	✓	-	-	-
Aircraft support centre, 2001 [216]	✓	-	-	-	-	-	-
Electrical parts, starters/generators, generator control units, fans, hydraulics, wheels, and breaks assembly shops, 2001 [217]	✓	-	-	-	-	-	-
Microwave popcorn production, 2001 [218]	-	-	-	-	✓	-	-
Flock production, 2000 [219]	✓	-	-	-	-	-	-
Beverage delivery company *, 2000 [220]	✓	-	-	-	-	✓	✓
Flat, clear glass producer*, 2000 [221]	-	-	-	✓	-	✓	✓
Automotive foam cushion manufacturing, 2000 [222]	-	-	-	✓	-	-	-
Flocking facility, 2000 [223]	✓	-	-	-	-	-	-
Aircraft engine facility, 2000 [224]	-	-	-	✓	-	-	-
Military aircraft manufacturer, 2000 [225]	✓	-	-	-	-	-	-
Backhoe, crawler dozers and rough terrain forklifts manufacturer, 2000 [226]	-	-	-	✓	-	-	-
Plastic injection-moulding facility, 2000 [227]	-	✓	-	-	-	-	-
Automobile transmission plant **, 2000 [228]	✓	-	-	✓	-	-	✓
Aircraft support centre, 2000 [229]		✓	-	-	-	-	-
Beef company, 2000 [230]	✓	-	-	-	-	-	-
Precious metal recycling facility, 2000 [231]	✓	-	-	-	-	-	-
Turkey processing plant, 2000 [232]	✓	-	-	-	-	-	-
Hydraulic commercial and industrial elevator production **, 2000 [233]	✓	-	-	✓	-	-	✓
Wire harness and heating, ventilation, and air conditioning components assembly shop, 2000 [234]	✓	-	-	-	-	-	-
Steel galvanizing operation, 2000 [235]	✓	-	-	-	-	-	-
Total (percentage)	87 (42%)	59 (28.5%)	3 (1.4%)	32 (15.5%)	5 (2.4%)	14 (6.8%)	7 (3.4%)

* Numeric count included as technical assistance only| ** Numeric count included as other.

**Table 2:** Quantitative presentation of the occupational hygiene measurement outcomes used during investigations.

Manufacturing Industry Type, Year (Reference)	Target Occupational Health Stressor	Measured Exposure Levels ^A^	Compliance to Health and Safety Standards				Complaint Justified
			SA	OSHA	NIOSH	HSE	
Coffee roasting, flavouring, and packaging facility, 2020 [28]	Diacetyl: Full-shift personal breathing zone (PBZ) samples	4.3–166	NR ^B^	NR	0.005(✕)	0.02(✕)	YES
	2,3-pentanedione: Full-shift PBZ samples	ND ^C^–199	NR	NR	NE^D^	NR	UNCLEAR
	Diacetyl: Full-shift PBZ samples	3.3–163.8	NR	NR	0.005(✕)	0.02(✕)	YES
	2,3-pentanedione: Full-shift PBZ samples	1.8–899.6	NR	NR	0.0093(✕)	NR	YES
	2,3-hexanedione: Full-shift PBZ samples	ND–15.7	NR	NR	NE	NR	UNCLEAR
Coffee roasting, flavouring, and packaging facility, 2020 [29]	Diacetyl: Full-shift PBZ samples	38.1–185.4	NR	NR	0.005(✕)	0.02(✕)	YES
	2,3-pentanedione: Full-shift PBZ samples	20.5–279.9	NR	NR	0.0093(✕)	NR	YES
	2,3-hexanedione: Full-shift PBZ samples	1.1–9.1	NR	NR	NE	NR	UNCLEAR
Architectural metal fabrication workshop, 2020 [30]	Chromium: Full-shift PBZ samples	ND–0.001	0.5(✓)	1000(✓)	500(✓)	0.5(✓)	YES
	Iron oxide: Full-shift PBZ samples	0.04–1.008	5(✓)	10(✓)	5(✓)	5(✓)	NO
	Manganese: Full-shift PBZ samples	0.002–0.022	5(✓)	5(✓)	1(✓)	0.5(✓)	NO
	Zinc oxide: Full-shift PBZ samples	ND–0.004	5(✓)	10(✓)	5(✓)	5(✓)	NO
Coffee roasting and packaging facility, 2019 [31]	Diacetyl: Full-shift PBZ samples	8.9–420.9	NR	NR	0.005(✕)	NR^e^	YES
	2,3-pentanedione: Full-shift PBZ samples	4.9–275.9	NR	NR	0.0093(✕)	NR	YES
Electronics recycling company, 2019 [32]	Iron: Full-shift PBZ samples	ND–19	5(✕)	10(✕)	5(✕)	5(✕)	YES
	Lead: Full-shift PBZ samples	ND–0.08	0.15(✓)	0.050(✕)	0.050(✕)	0.15(✕)	YES
	Manganese: Full-shift PBZ samples	ND–0.09	5(✓)	5(✓)	1(✓)	0.5(✓)	NO
	Nickel: Full-shift PBZ samples	ND–0.46	0.1(✕)	1(✓)	0.015(✕)	0.5(✓)	YES
	Zinc: Full-shift PBZ samples	ND–7.8	5(✕)	10(✓)	5(✕)	5(✕)	YES
	Noise (Peak noise): Area noise levels	117–123	NR	140(✓)	140(✓)	140(✓)	YES
Coffee roasting and packaging facility and two off-site retail cafes, 2019 [33]	Diacetyl: Full-shift PBZ samples	0.7–13.9	NR	NR	0.005(✕)	0.02(✕)	YES
	2,3-pentanedione: Full-shift PBZ samples	<0.5–15.6	NR	NR	0.0093(✕)	NR	YES
	2,3-hexanedione: Full-shift PBZ samples	<0.5 - <0.6	NR	NR	NE	NR	UNCLEAR
Rubber manufacturing facility, 2019 [34]	Total volatile organic compounds: Spot measurements	0.647 - 8	NR	NR	NE	NR	UNCLEAR
	Carbon monoxide: Spot measurements	0.9–6.4	50(✓)	50(✓)	35(✓)	20(✓)	NO
Paper converting equipment manufacturing facility, 2019 [35]	Thoracic particle mass: Full-shift PBZ samples	ND–1.58	10(✓)	15(✓)	10(✓)	10(✓)	NO
	Metalworking fluid: Full-shift PBZ samples	ND–0.32	NR	NR	0.40(✓)	NR	NO
Coffee roasting, flavouring, and packaging facility, 2019 [36]	Diacetyl: Full-shift PBZ samples	ND–1.3	NR	NR	0.005(✕)	0.02(✕)	YES
	2,3-pentanedione: Full-shift PBZ samples	ND–1.6	NR	NR	0.0093(✕)	NR	YES
	2,3-hexanedione: Full-shift PBZ samples	ND	NR	NR	NE	NR	UNCLEAR
Aircraft power plant parts manufacturer, 2019 [38]	Chromium: Full-shift PBZ samples	0.0013–0.012	0.5(✓)	1(✓)	0.5(✓)	0.5(✓)	NO
	Hexavalent chromium: Full-shift PBZ samples	ND–0.000001	0.05(✓)	0.1(✓)	1(✓)	0.05(✓)	NO
	Nickel: Full-shift PBZ samples	ND–0.035	1(✓)	1(✓)	0.015(✕)	0.1(✓)	YES
	Noise: Employee full-shift noise exposure	50.3–88.2 ^E^ 76–95 ^F^	85(✕)	85(✕) 90(✕)	85(✕)	80(✓) 87(✕)	YES
Precast concrete manufacturer, 2019 [39]	Noise: employee noise exposures: full-shift noise exposure	73.1–90.2 ^E^ 79–95.1 ^F^	85(✕)	85(✕) 90(✕)	85(✕)	80(✓) 87(✕)	YES
Ceramic tile manufacturer, 2019 [41]	Sulphuric acid: Full-shift PBZ samples	0.0022–0.012	1	1	1	0.05	NO
	Heat stress: area measurements	25.7–29.5	30 ^G^(✕)	NR	26.7 ^G^(✕)	Varies	YES
Optical media production company, 2018 [42]	2-Butoxyethanol: Full-shift PBZ samples	0.001–0.1	25(✓)	50(✓)	5(✓)	25(✓)	NO
	Ethylbenzene: Full-shift PBZ samples	0.0006–0.01	100(✓)	100(✓)	100(✓)	100(✓)	NO
	Naphthalene: Full-shift PBZ samples	0.001–0.1	10(✓)	10(✓)	10(✓)	NR	NO
	Trimethylbenzene: Full-shift PBZ samples	0.001–0.1	25(✓)	NR	25(✓)	25(✓)	NO
	Xylene: Full-shift PBZ samples	0.001–0.1	100(✓)	100(✓)	100(✓)	50(✓)	NO
Coffee roasting and packaging facility, 2018 [43]	Diacetyl: Full-shift PBZ samples	ND–20.7	NR	NR	0.005(✕)	0.02(✕)	YES
	2,3-pentanedione: Full-shift PBZ samples	0.2–24	NR	NR	0.0093(✕)	NR	YES
	2,3-hexanedione: Full-shift PBZ samples	ND–0.5	NR	NR	NE	NR	UNCLEAR
Coffee roasting and packaging facility, 2018 [44]	Diacetyl: Full-shift PBZ samples	0.6–2.9	NR	NR	0.005(✕)	0.02(✕)	YES
	2,3-pentanedione: Full-shift PBZ samples	0.4–2.6	NR	NR	0.0093(✕)	NR	YES
	2,3-hexanedione: Full-shift PBZ samples	ND	NR	NR	NE	NR	UNCLEAR
Coffee roasting and packaging facility, 2018 [45]	Diacetyl: Full-shift PBZ samples	0.9–4.7	NR	NR	0.005(✕)	0.02(✕)	YES
	2,3-pentanedione: Full-shift PBZ samples	0.9–3.9	NR	NR	0.0093(✕)	NR	YES
	2,3-hexanedione: Full-shift PBZ samples	ND	NR	NR	NE	NR	NO
Steel coil pickling plant, 2018 [46]	Oil mist: Full-shift PBZ samples	0.053–0.081	NR	5(✓)	5(✓)	NR	NO
	Noise: Full-shift personal noise exposures	70.3–83.6 ^E^ 83.9–90.5 ^F^	85(✕)	85(✕) 90(✕)	85(✕)	80(✕) 87(✕)	YES
	Oil mist: Full-shift area air samples	ND–0.088	NR	5(✓)	5(✓)	NR	NO
	Noise: Area noise measurements	50.2–90.3 ^E^ 76–93.3 ^F^	85(✕)	85(✕) 90(✕)	85(✕)	80(✕) 87(✕)	YES
Bullet manufacturer, 2018 [49]	Lead: Full-shift PBZ samples	0.0023–0.02	0.15(✓)	0.050(✓)	0.050(✓)	0.15(✓)	NO
	Tin: Full-shift PBZ samples	ND–0.0009	2(✓)	2(✓)	2(✓)	2(✓)	NO
	Noise: Spot measurements	85–99 ^F^	85(✕)	85(✕) 90(✕)	85(✕)	80(✕) 87(✕)	YES
Engine machining plant, 2018 [50]	Metalworking fluid: Full-shift PBZ samples	ND–0.17	NR	NR	0.40(✓)	NR	NO
Coffee roasting and packaging facility, 2018 [53]	Diacetyl: Full-shift PBZ samples	7.4–40.5	NR	NR	0.005(✕)	0.02(✕)	YES
	2,3-pentanedione: Full-shift PBZ samples	4.8–27.1	NR	NR	0.0093(✕)	NR	YES
	2,3-hexanedione: Full-shift PBZ samples	ND–1.3	NR	NR	NE	NR	UNCLEAR
Coffee roasting and packaging facility, 2018 [54]	Diacetyl: Full-shift PBZ samples	4.8–33.3	NR	NR	0.005(✕)	0.02(✕)	YES
	2,3-pentanedione: Full-shift PBZ samples	2.2–177.9	NR	NR	0.0093(✕)	NR	YES
	2,3-hexanedione: Full-shift PBZ samples	ND–2	NR	NR	NE	NR	UNCLEAR
Two coffee roasting and packaging facility, 2018 [55]	Diacetyl: Full-shift PBZ samples	2.3–9.4	NR	NR	0.005(✕)	0.02(✕)	YES
	2,3-pentanedione: Full-shift PBZ samples	1.3–5.3	NR	NR	0.0093(✕)	NR	YES
	2,3-hexanedione: Full-shift PBZ samples	ND–0.7	NR	NR	NE	NR	UNCLEAR
Coffee roasting and packaging facility, 2018 [56]	Diacetyl: Full-shift PBZ samples	0.7–5.6	NR	NR	0.005(✕)	0.02(✕)	YES
	2,3-pentanedione: Full-shift PBZ samples	0.6–33	NR	NR	0.0093(✕)	NR	YES
	2,3-hexanedione: Full-shift PBZ samples	ND	NR	NR	NR	NR	UNCLEAR
Coffee roasting and packaging facility, 2018 [57]	Diacetyl: Full-shift PBZ samples	0.5–21.5	NR	NR	0.005(✕)	0.02(✕)	YES
	2,3-pentanedione	ND–15.8	NR	NR	0.0093(✕)	NR	YES
	2,3-hexanedione	ND–0.4	NR	NR	NE	NR	UNCLEAR
Coffee roasting and packaging facility, 2018 [58]	Diacetyl: Full-shift personal breathing zone samples	0.5–25.6	NR	NR	0.005(✕)	0.02(✕)	YES
	2,3-pentanedione: Full-shift PBZ samples	ND–15.8	NR	NR	0.0093(✕)	NR	YES
	2,3-hexanedione: Full-shift PBZ samples	ND–0.4	NR	NR	NE	NR	UNCLEAR
3-D printing product manufacturing facility, 2017 [59]	Acetone: Full-shift PBZ samples	0.05–0.11	750(✓)	1000(✓)	250(✓)	500(✓)	NO
	Ethanol: Full-shift PBZ samples	0.05	1000(✓)	1000(✓)	1000(✓)	1000(✓)	NO
	Isopropyl alcohol: Full-shift PBZ samples	2–2.6	400(✓)	400(✓)	400(✓)	400(✓)	NO
	m,p-Xylene: Full-shift PBZ samples	0.002–0.0005	100(✓)	100(✓)	100(✓)	50(✓)	NO
Aircraft equipment depot, 2017 [60]	Cadmium (total particulate): Full-shift PBZ samples	ND–00000093	0.05(✓)	0.005(✓)	LFL^H^	0.025(✓)	NO
	Cadmium (respirable particulate): Full-shift PBZ samples	ND–0.00000027	0.05(✓)	0.005(✓)	LFL^H^	0.025(✓)	NO
Plastic film assembly facility, 2017 [61]	Acetaldehyde: Full-shift PBZ samples	0.0064–0.026	100(✓)	200(✓)	NE	20(✓)	NO
	Formaldehyde: Full-shift PBZ samples	0.0046–0.068	2(✓)	2(✓)	0.1(✓)	2(✓)	NO
	Respirable dust: Full-shift PBZ samples	0.17–0.49	5(✓)	5(✓)	5(✓)	4(✓)	NO
Water heater manufacturing, 2017 [62]	Respirable dust: Full-shift PBZ samples	0.000063–0.00059	5(✓)	5(✓)	5(✓)	4(✓)	NO
	Crystalline silica: Full-shift PBZ samples (respirable)	0.000011–0.000104	0.04(✓)	0.05(✓)	0.05(✓)	0.1(✓)	NO
	Manganese: Full-shift PBZ samples	0.00000056–0.00003	5(✓)	5(✓)	1(✓)	0.05(✓)	NO
	Iron oxide: Full-shift PBZ samples	0.000033–0.00024	5(✓)	10(✓)	5(✓)	5(✓)	NO
	MDI monomer: Full-shift PBZ samples	ND–0.00000007	0.2(✓)	0.02(✓)	0.005(✓)	0.02(✓)	NO
Coffee processing facility, 2017 [63]	Diacetyl: Full-shift PBZ samples	ND–7.2	NR	NR	0.005(✕)	0.02(✕)	YES
	2,3-pentanedione: Full-shift PBZ samples	ND–6.9	NR	NR	0.0093(✕)	NR	YES
	2,3-hexanedione: Full-shift PBZ samples	ND	NR	NR	NR	NR	UNCLEAR
Coffee roasting and packaging facility and attached retail café, 2017 [64]	Diacetyl: Full-shift PBZ samples	ND–5.9	NR	NR	0.005(✕)	0.02(✕)	YES
	2,3-pentanedione: Full-shift PBZ samples	0.9–5.2	NR	NR	0.0093(✕)	NR	YES
	2,3-hexanedione: Full-shift PBZ samples	ND	NR	NR	NR	NR	UNCLEAR
Coffee processing plant, 2017 [65]	Diacetyl: Full-shift PBZ samples	1.3–4.1	NR	NR	0.005(✕)	0.02(✕)	YES
	2,3-pentanedione: Full-shift PBZ samples	0.9–4.9	NR	NR	0.0093(✕)	NR	YES
	2,3-hexanedione: Full-shift PBZ samples	ND	NR	NR	NE	NR	UNCLEAR
Grey and ductile iron foundry, 2017 [66]	Noise: Personal noise exposure	95.7–107.5 ^E^ 97.7–107.9 ^F^	85(✕)	85(✕) 90(✕)	85(✕)	80(✕) 87(✕)	YES
	Noise: Area noise levels and spectral analysis	93–120 ^F^	85(✕)	85(✕) 90(✕)	85(✕)	80(✕) 87(✕)	YES
Coffee roasting and packaging facility, 2017 [67]	Diacetyl: Full-shift PBZ samples	2.8–18.8	NR	NR	0.005(✕)	0.02(✕)	YES
	2,3-pentanedione: Full-shift PBZ samples	2.9–18.7	NR	NR	0.0093(✕)	NR	YES
	2,3-hexanedione: Full-shift PBZ samples	ND	NR	NR	NE	NR	UNCLEAR
Coffee roasting and packaging facility [68]	Diacetyl: Full-shift PBZ samples	ND–13.1	NR	NR	0.005(✕)	0.02(✕)	YES
	2,3-pentanedione: Full-shift PBZ samples	ND–7.5	NR	NR	0.0093(✕)	NR	YES
	2,3-hexanedione: Full-shift PBZ samples	ND–0.4	NR	NR	NE	NR	UNCLEAR
Poultry production plant, 2017k [69]	Peracetic acid: Full-shift PBZ samples	0.0080–0.0092	NR	NR	NR	NR	UNCLEAR
	Hydrogen peroxide: Full-shift PBZ samples	0.027–0.028	1(✓)	1(✓)	1(✓)	1(✓)	YES
	Acetic acid: Full-shift PBZ samples	0.047–0.078	10(✓)	10(✓)	10(✓)	10(✓)	YES
Poultry production plant, 2016 [70]	Peracetic acid: Full-shift PBZ samples	ND	NR	NR	NR	NR	NO
	Hydrogen peroxide: Full-shift PBZ samples	ND	1(✓)	1(✓)	1(✓)	1(✓)	YES
	Acetic acid: Full-shift PBZ samples	0.030	10(✓)	10(✓)	10(✓)	10(✓)	NO
Stone countertop manufacturing plant, 2016 [71]	Respirable dust: Full-shift PBZ samples	ND–0.00038	5(✓)	5(✓)	5(✓)	4(✓)	NO
	Crystalline silica: Full-shift PBZ samples	ND–0.0013	0.04(✓)	0.05(✓)	0.05(✓)	0.1(✓)	NO
Hammer forge company, 2016 [72]	Noise: Full-shift TWA noise exposures	65.2–107 ^E^ 83.4–110.4 ^F^	85(✕)	85(✕) 90(✕)	85(✕)	80(✕) 87(✕)	YES
	Noise: Impact noise levels of forge equipment	118–148 ^F^	NR	NR	137(✕)	137(✕)	YES
	Whole body vibration: Hammers	0.02–0.56	NR	NR	NR	1.15(✓)	NO
	Harm-arm vibration: Grinders	0.50–4.40	NR	NR	NR	5(✓)	NO
	Heat: General	22–33.9	30 ^G^(✕)	NR	26.7 ^G^(✕)	Varies	YES
Riffle barrel manufacturing, 2016 [73]	Metalworking fluid mist (thoracic particulate): Full-shift PBZ samples	0.12–0.4	NR	NR	0.40(✓)	NR	NO
	Metalworking fluid mist (extracted MWF particulate): Full-shift PBZ samples	0.09–0.34	NR	NR	NE	NR	UNCLEAR
Security portal manufacturer, 2016 [74]	Chromium: Full-shift PBZ samples	ND–0.0005	0.5(✓)	1(✓)	0.5(✓)	0.5(✓)	YES
	Manganese: Full-shift PBZ samples	0.00001–0.0014	5(✓)	5(✓)	1(✓)	0.05(✓)	YES
	Nickel: Full-shift PBZ samples	ND–0.0002	1(✓)	1000(✓)	15(✓)	0.5(✓)	YES
	Noise: Personal noise sampling	66.2–89.5 ^E^	85(✕)	85(✕) 90(✓)	85(✕)	80(✓) 87(✕)	YES
Automobile parts manufacturing plant, 2016 [75]	Noise: Personal noise exposure	53.2–68.9 ^E^ 77.9–84.5 ^E^	85(✕)	85(✕) 90(✓)	85(✕)	80(✓) 87(✕)	YES
	2-butoxyethanol: Full-shift PBZ samples	0.013–0.023	25(✓)	50(✓)	5(✓)	25(✓)	NO
	Isopropyl alcohol: Full-shift PBZ samples	4.1–5	400(✓)	400(✓)	400(✓)	400(✓)	NO
	Pentane: Full-shift PBZ samples	0.062–0.067	600(✓)	1000(✓)	120(✓)	600(✓)	NO
	Toluene: Full-shift PBZ samples	0.038–0.046	50(✓)	200(✓)	100(✓)	50(✓)	NO
Coal and copper slag processing facility, 2016 [78]	Total dust: Full-shift PBZ samples	0.12–6.56	10(✓)	15(✓)	10(✓)	10(✓)	NO
	Respirable dust: Full-shift PBZ samples	ND–0.70	5(✓)	5(✓)	5(✓)	4(✓)	NO
	Crystalline silica: Full-shift PBZ samples	ND–0.42	0.04(✕)	0.05(✕)	0.05(✕)	0.1(✕)	YES
	Chromium: Full-shift PBZ samples	0.0003–0.0014	0.5(✓)	0.5(✓)	1(✓)	0.5(✓)	NO
	Copper: Full-shift PBZ samples	ND–0.479	1(✓)	1(✓)	1(✓)	1(✓)	NO
	Tin: Full-shift PBZ samples	0.0004–0.059	2(✓)	2(✓)	2(✓)	2(✓)	NO
Fiberglass-reinforced wind turbine blade manufacturing, 2016 [80]	Styrene: Full-shift PBZ samples	0.091–56	100(✓)	100(✓)	50(✕)	100(✓)	NO
	Total dust: Full-shift PBZ samples	0.28–90	10(✕)	15(✕)	10(✕)	10(✕)	YES
Automotive engine water pump manufacturer, 2016 [81]	Metalworking fluid mist (thoracic particulate): Full-shift PBZ samples	0.19–0.76	NR	NR	0.40(✕)	NR	NO
	Formaldehyde: Full-shift PBZ samples	0.041–0.19	2(✓)	0.75(✓)	0.016(✕)	2(✓)	NO
Garlic paste production process, 2015 [82]	Diallyl disulphide: Full-shift PBZ samples	0.09–0.63	NR	2(✓)	2(✓)	NR	NO
Aircraft ejection seat manufacturer, 2015 [83]	Metalworking fluid mist (thoracic particulate): Full-shift PBZ samples	0.08–0.20	NR	NR	0.40(✓)	NR	NO
Grey and ductile iron foundry, 2015 [85]	Noise: Personal noise measurements	91.3–103.7 ^E^ 94.2–105.9 ^F^	85(✕)	85(✕) 90(✕)	85(✕)	80(✕) 87(✕)	YES
	Noise: Area measurements	105–114 ^E^ 100–103 ^F^	85(✕)	85(✕) 90(✕)	85(✕)	80(✕) 87(✕)	YES
Dry cleaning shop, 2015 [86]	Butylal: Full-shift PBZ samples	0.14–0.83	NR	NR	NR	NR	UNCLEAR
	Butylal: Task-based breathing zone samples	0.57–1.9	NR	NR	NR	NR	UNCLEAR
	Butylal: Full-shift PBZ samples	0.18–0.19	NR	NR	NR	NR	UNCLEAR
Orthopaedic implant manufacturer, 2015 [87]	Hexavalent chromium: Full-shift PBZ samples	ND–0.0001	0.05(✓)	0.1(✓)	0.005(✓)	0.05(✓)	NO
	Hexavalent chromium: Area samples	ND–0.000001	0.05(✓)	0.1(✓)	0.005(✓)	0.01	NO
	Metalworking fluid: Full-shift PBZ samples	ND	NR	NR	0.4(✓)	NR	NO
	Metalworking fluid (total particulate): Area samples	ND	NR	NR	0.4(✓)	NR	NO
	Total particulate in air: Full-shift PBZ samples	0.069–21	10(✕)	15(✕)	10(✕)	10(✕)	YES
	Noise: Personal noise monitoring	67–93 ^F^	85(✕)	85(✕) 90(✕)	85(✕)	80(✓) 87(✕)	YES
Polymer additive manufacturing facility, 2014 [89]	Aniline: Full-shift PBZ samples	ND	2(✓)	5(✓)	LFL^I^(✓)	1(✓)	NO
	Hydrogen sulphide: Full-shift PBZ samples	ND	10(✓)	20(✓)	10(✓)	5(✓)	NO
	OTOS dust: Full-shift PBZ samples	0.91–1.4	10(✓)	15(✓)	10(✓)	NR	NO
Electrical cables accessories manufacturing, 2014 [93]	Formaldehyde: Full-shift PBZ samples	0.0032–0.006	2(✓)	0.75(✓)	0.016(✓)	2(✓)	NO
	Toluene: Full-shift PBZ samples	2.3–13	50(✓)	200(✓)	100(✓)	50(✓)	NO
	Ethylbenzene: Full-shift PBZ samples	0.25–1.2	100(✓)	100(✓)	100(✓)	100(✓)	NO
	Xylene: Full-shift PBZ samples	0.8–5.4	100(✓)	100(✓)	100(✓)	50(✓)	NO
Automotive lead-acid battery recycling company, 2014 [95]	Lead: Full-shift PBZ samples	0.004–4.1	0.15(✕)	0.050(✕)	0.050(✕)	0.15(✕)	YES
	Noise: Personal noise exposure	69–86 ^E^ 82–92 ^F^	85(✕)	85(✕) 90(✕)	85(✕)	80(✕) 87(✕)	YES
	Heat: Area WBGT measurements	15.6–31	30 ^G^(✕)	NR	26.7 ^G^(✕)	Varies	YES
Furniture manufacturing plant, 2013 [97]	Isobutyl acetate: Full-shift PBZ samples	0.31–0.43	200(✓)	150(✓)	150(✓)	200(✓)	NO
	nButyl acetate: Full-shift PBZ samples	0.055–0.42	150(✓)	150(✓)	150(✓)	150(✓)	NO
	2-Propoxyethanol: Full-shift PBZ samples	0.083–0.11	NR	NR	NR	NR	UNCLEAR
	2-Butoxyethanol: Full-shift PBZ samples	ND–0.007	25(✓)	50(✓)	5(✓)	25(✓)	NO
Cream cheese manufacturing facility, 2013 [99]	Diacetyl: Full-shift PBZ samples	0.4–15.1	NR	NR	0.005(✕)	0.02(✕)	YES
	2,3-pentanedione: Full-shift PBZ samples	ND	NR	NR	0.0093(✓)	NR	NO
	Acetoin: Full-shift PBZ samples	1.7–85.1	NR	NE	NE	NR	UNCLEAR
Snack food production facility, 2013 [100]	Sodium hydroxide: 8-hour TWA air concentration	0.01	2(✓)	2(✓)	2(✓)	2(✓)	NO
Poultry breading plant, 2013 [102]	Inhalable flour dust: Full-shift PBZ samples	0.22–93	10(✕)	15(✕)	NE	10(✕)	YES
	Inhalable wheat: Full-shift PBZ samples	ND–0.44	10(✓)	10(✓)	4(✓)	10(✓)	NO
	Inhalable soy: Full-shift PBZ samples	ND–0.00001	10(✓)	10(✓)	4(✓)	10(✓)	NO
Aluminium beverage can manufacturing, 2012 [104]	Noise: Area noise levels and spectral analysis	100.5–114 ^E^	85(✕)	85(✕) 90(✕)	85(✕)	80(✕) 87(✕)	YES
	Noise: Full-shift personal noise exposure	71.2–100.2 ^E^ 84.2–102.7 ^F^	85(✕)	85(✕) 90(✕)	85(✕)	80(✕) 87(✕)	YES
	Metalworking fluid: Full-shift PBZ samples	0.09–0.28	NR	NR	0.40(✓)	NR	NO
	Hydrofluoric acid: Full-shift PBZ samples	0.0096	3(✓)	2.5(✓)	2.5(✓)	1.8(✓)	NO
	Hydrofluoric acid: Area air samples	0.005–0.24	3(✓)	2.5(✓)	2.5(✓)	1.8(✓)	NO
	Dibutylaminoethanol: Full-shift PBZ samples	0.20–0.26	NR	NR	14 (✓)	NR	NO
	Dibutylaminoethanol: Area air samples	0.11–0.28	NR	NR	14 (✓)	NR	NO
	Formaldehyde: Full-shift PBZ samples	0.020–0.090	2(✓)	0.75(✓)	0.016(✓)	2(✓)	NO
	Formaldehyde: Area air samples	0.006–0.040	2(✓)	0.75(✓)	0.016(✕)	2(✓)	YES
Poultry processing facility, 2012 [108]	Soluble chlorine: Full-shift PBZ samples	ND–0.00013	0.5(✓)	1(✓)	0.5(✓)	0.5(✓)	NO
	Trichloramine: Full-shift PBZ samples	ND–0.000045	NR	NR	NE	NR	UNCLEAR
Aircraft engine manufacturing facility, 2012 [110]	Metalworking fluid mist (thoracic particulate): Full-shift PBZ samples	ND–0.29	NR	NR	0.40(✓)	NR	NO
	Metalworking fluid mist (extracted MWF particulate): Full-shift PBZ samples	ND–0.31	NR	NR	NE	NR	UNCLEAR
Drum refurbishing plant, 2011 [112]	Cumene: Work-shift PBZ samples	0.007–0.7	25(✓)	50(✓)	50(✓)	25(✓)	NO
	Toluene: Work-shift PBZ samples	ND–0.35	50(✓)	200(✓)	100(✓)	50(✓)	NO
	Trimethyl benzenes: Work-shift PBZ samples	0.47–30.51	25(✓)	NR	25(✕)	25(✓)	YES
	Xylene: Work-shift PBZ samples	0,0168–1.52	50(✓)	400(✓)	100(✓)	50(✓)	NO
	Noise: Personal work-shift TWA noise exposure measurements	81.3–104.9 ^E^	85(✕)	85(✕) 90(✓)	85(✕)	80(✕) 87(✕)	YES
Ink ribbon manufacturing, 2011 [113]	Methyl ethyl ketone: Area samples	0.12–85	200(✓)	200(✓)	200(✓)	NR	NO
	Xylene (para): Area samples	ND–0.049	100(✓)	100(✓)	100(✓)	50(✓)	NO
	Toluene: Area samples	0.34–11	50(✓)	100(✓)	200(✓)	50(✓)	NO
Aluminium smelter, 2011 [114]	Heat stress: Area measurements	26–48.9	30 ^G^(✕)	NR	26.7 ^G^(✕)	Varies	YES
Semiconductor manufacturing plant, 2011 [116]	Carbon monoxide: Full-shift PBZ samples	0–375	50(✓)	50(✓)	35(✓)	20(✓)	NO
Immortalis Botanicals, 2010 [117]	Toluene: TWA PBZ sample	0.064–0.069	50(✓)	200(✓)	100(✓)	50(✓)	NO
Steel manufacturing, 2010 [118]	Carbon monoxide: Full-shift PBZ samples	3–7	50(✓)	50(✓)	35(✓)	20(✓)	NO
	Lead: Full-shift PBZ samples	ND–0.0088	0.15(✓)	0.050(✓)	0.050(✓)	0.15(✓)	NO
	Iron: Full-shift PBZ samples	0.042–2.3	5(✓)	10(✓)	5(✓)	5(✓)	NO
Electrolytic manganese dioxide processing plant, 2010 [120]	Manganese: Full-shift PBZ samples	0.015–1.6	5(✓)	5(✓)	1(✕)	0.5(✕)	YES
Aircraft manufacturing plant, 2010 [121]	Total dust: Full-shift PBZ samples	ND–0.28	10(✓)	15(✓)	NE	10(✓)	NO
	Respirable dust: Full-shift PBZ samples	ND–0.29	5(✓)	5(✓)	5(✓)	4(✓)	NO
Road markings manufacturing, 2009 [123]	Respirable dust: Full-shift PBZ samples	ND–0.18	5(✓)	5(✓)	5(✓)	4(✓)	NO
	Formaldehyde: Full-shift PBZ samples	ND–0.0098	2(✓)	0.75(✓)	0.016(✓)	2(✓)	NO
Road sign printing, 2009 [124]	Toluene: Full-shift PBZ samples	9.8–17	50(✓)	200(✓)	100(✓)	50(✓)	NO
	n-Hexane: Full-shift TWA PBZ samples	2.9–5.7	20(✓)	500(✓)	50(✓)	20(✓)	NO
	Isopropyl alcohol: Full-shift TWA PBZ samples	6.9–10	400(✓)	400(✓)	400(✓)	400(✓)	NO
	Acetone: Full-shift TWA PBZ samples	14–31	750(✓)	1000(✓)	250(✓)	500(✓)	NO
	Cyclohexanone: Full-shift TWA PBZ samples	0.28–0.60	50(✓)	50(✓)	25(✓)	10(✓)	NO
Metal furniture manufacturing, 2009 [125]	Welding fumes: Manganese: Full-shift PBZ samples	0.81–70	1(✕)	5(✕)	1(✕)	0.5(✕)	YES
	Welding fumes: Iron: Full-shift PBZ samples	34–1830	5(✕)	10(✕)	5(✕)	5(✕)	YES
	Respirable dust: Full-shift PBZ samples	ND–8.4	5(✕)	5(✕)	5(✕)	NR	YES
	Total dust: Full-shift PBZ samples	0.80 - 130	10(✕)	15(✕)	10(✕)	10(✕)	YES
Printed circuit board manufacturing, 2009 [126]	Toluene: Full-shift PBZ samples	0.17–3	50(✓)	200(✓)	100(✓)	50(✓)	NO
	Xylene: Full-shift PBZ samples	0.063–4	100(✓)	100(✓)	100(✓)	50(✓)	NO
	n-Butyl acetate: Full-shift PBZ samples	0.99–40	150(✓)	150(✓)	150(✓)	150(✓)	NO
	MEK: Full-shift PBZ samples	0.045–4.7	200(✓)	200(✓)	200(✓)	200(✓)	NO
	2-Butoxyethanol: Full-shift PBZ samples	0.0062–0.0095	25(✓)	50(✓)	5(✓)	25(✓)	NO
	Benzyl alcohol: Full-shift PBZ samples	0.57–2.6	NR	1(✕)	1(✕)	NR	YES
	Noise: Full-shift noise exposure doses	20–66.4 ^F^	85(✓)	85(✓) 90(✓)	85(✓)	80(✓) 87(✓)	NO
Bakery, 2009 [127]	Flour dust: Inhalable Full-shift PBZ samples	ND–65	10(✕)	15(✕)	NE	10(✕)	YES
	α-amylase: Full-shift PBZ samples	ND–11	NR	NR	NE	NR	UNCLEAR
	Wheat: Full-shift PBZ samples	ND–900	10(✕)	10(✕)	4(✕)	10(✕)	YES
Flavourings, modified dairy products, and bacterial additive manufacturing, 2009 [128]	Diacetyl: Full-shift PBZ samples	ND–4.30	NR	NR	0.0005(✕)	0.02(✕)	YES
	Acetaldehyde: Full-shift PBZ samples	ND	100(✓)	200(✓)	LFC	20(✓)	NO
	Respirable dust: Full-shift PBZ samples	ND–1.25	5(✓)	5(✓)	5(✓)	4(✓)	NO
Tungsten carbide manufacturing, 2009 [129]	Cobalt: Full-shift PBZ samples	0.0016–0.815	0.1(✕)	0.1(✕)	0.05(✕)	0.1(✕)	YES
	Chromium: Full-shift PBZ samples	ND–0.0029	0.5(✓)	1(✓)	0.5(✓)	0.5(✓)	NO
	Nickel: Full-shift PBZ samples	0.0002–0.805	0.5(✕)	1(✓)	0.015(✕)	0.5(✕)	YES
	Total dust: Full-shift PBZ samples	0.0217–10.86	10(✕)	15(✓)	NE	10(✕)	YES
	Metalworking fluid: Full-shift PBZ samples	0.0001–0.0009	NR	NR	0.40(✓)	NR	NO
Three commercial kitchens, 2009 [130]	Diacetyl, acetoin, nitrogen dioxide: Full-shift PBZ samples	ND	-	-	-	-	NO
Automotive parts manufacturing, 2008 [131]	Heat stress: Area WBGT index	21.1–25.6	30 ^G^(✓)	NR	26.7 ^G^(✓)	Varies	YES
	Noise: Area noise levels	90–100 ^E^	85(✕)	85(✕) 90(✕)	85(✕)	80(✕) 87(✕)	YES
Pottery shop, 2008 [136]	Respirable particulates: Full-shift PBZ samples	0.15–0.34	5(✓)	5(✓)	5(✓)	4(✓)	NO
	Silica: Full-shift PBZ samples	ND	0.04(✓)	0.05(✓)	0.05(✓)	0.1(✓)	NO
	Respirable particulates: Task-based PBZ samples	0.43–2.4	5(✓)	5(✓)	5(✓)	4(✓)	NO
	Silica: Task-based PBZ samples	ND–1.3	0.04(✓)	0.05(✓)	0.05(✓)	0.1(✓)	NO
Entek manufacturing, 2008 [137]	Trichloroethylene: Full-shift PBZ samples	1.7 - 130	100(✕)	100(✕)	25(✕)	100(✕)	YES
	Noise: Area noise levels	75–97 ^E^	85(✕)	85(✕) 90(✕)	85(✕)	80(✕) 87(✕)	YES
	Noise: Noise dose levels	20–93.2 ^E^ 84.3–104.6 ^F^	85(✕)	85(✕) 90(✕)	85(✕)	80(✕) 87(✕)	YES
Metal conduit manufacturing, 2008 [138]	Noise: Personal noise dosimetry measurements	72.2–95.6 ^E^ 81.7–102.7 ^E^	85(✕)	85(✕) 90(✕)	85(✕)	80(✕) 87(✕)	YES
	Metalworking fluids: Full-shift PBZ samples(thoracic part mass)	0.17–0.5	NR	NR	0.40(✕)	NR	YES
	Metalworking fluids: Full-shift PBZ samples (extracted MWF)	ND–0.32	NR	NR	NE	NR	UNCLEAR
	Acids: nitric acid: Full-shift PBZ samples	ND–0.054	2(✓)	5(✓)	5(✓)	1(✓)	NO
	Chromium VI: Full-shift PBZ samples	0.026–0.040	0.05(✓)	5(✓)	1(✓)	0.05(✓)	NO
	Welding fumes: Zinc: Full-shift PBZ samples	7.7–1450	5(✕)	5(✕)	5(✕)	NR	YES
	Welding fumes: Iron: Full-shift PBZ samples	11–380	5(✕)	10(✕)	5(✕)	5(✕)	YES
	Heat stress: Area measurements	26.2–30.5	30 ^G^(✕)	NR	26.7 ^G^(✕)	Varies	YES
	Noise: Area noise levels	81–96.5 ^F^	85(✕)	85(✕) 90(✕)	85(✕)	80(✕) 87(✕)	YES
Flavouring manufacturing plant, 2008 [139]	Acetoin: PBZ task-based samples	0.05–1.05	NR	NR	NE	NR	UNCLEAR
	Diacetyl: PBZ task-based samples	0.05–11.04	NR	NR	0.005(✕)	0.02(✕)	YES
	2-Furaldehyde: PBZ task-based samples	0.01–0.04	2(✓)	5(✓)	LFL	2(✓)	NO
	Acetaldehyde: PBZ task-based samples	0.19–4.02	100(✓)	200(✓)	LFL	20(✓)	NO
	Acetic acid: PBZ task-based samples	1.93	10(✓)	10(✓)	10(✓)	10(✓)	NO
	Butyric acid: PBZ task-based samples	1.20	NR	NR	NE	NR	UNCLEAR
	Propionic acid: PBZ task-based samples	1.43	10(✓)	NR	10(✓)	10(✓)	UNCLEAR
Glass bottle manufacturing, 2007 [140]	Heat stress: Area WBGT measurements	18.1–30.7	30 ^G^(✕)	NR	26.7 ^G^(✕)	Varies	YES
Specialty steel manufacturing, 2007 [144]	Noise: Noise dose levels	50–80.3 ^E^ 83.4–96 ^F^	85(✕)	85(✕) 90(✕)	85(✕)	80(✕) 87(✕)	YES
	Hydrochloric acid: Full-shift PBZ samples	ND–11	5(✕)	7(✕)	7(✕)	2(✕)	YES
	Sulphuric acid: Full-shift PBZ samples	ND–0.23	1(✓)	1(✓)	1(✓)	0.05(✕)	YES
	Metalworking fluid: Full-shift PBZ samples	0.57–2.6	NR	NR	0.40(✕)	NR	YES
	Oil mist: Full-shift PBZ samples	0.30–2.3	NR	5(✓)	5(✓)	NR	NO
Communications company, 2007 [145]	Noise: Area noise levels	52.8–69.9	85(✓)	85(✓) 90(✓)	85(✓)	80(✓) 87(✓)	NO
Poultry processing facility, 2007 [146]	Trichloramine: Full-shift PBZ samples	0.00006–0.00021	NR	NR	NE	NR	UNCLEAR
	Soluble chlorine: Full-shift PBZ samples	ND–0.0001	0.5(✓)	1(✓)	0.5(✓)	0.5(✓)	NO
Flavouring manufacturing plant, 2007 [149]	Diacetyl: Full-shift PBZ TWA samples	0.001–8.66	NR	NR	0.005(✕)	0.02(✕)	YES
	Acetoin: Full-shift PBZ TWA samples	0.002–0.894	NR	NE	NE	NR	UNCLEAR
	Acetaldehyde: Full-shift PBZ TWA samples	0.0001–0.185	100(✓)	200(✓)	LFL	20(✓)	NO
	Benzaldehyde: Full-shift PBZ TWA samples	0.0002–2.23	NR	NR	NE	NR	UNCLEAR
Ballistic systems manufacturing, 2006 [150]	Silver iodide: Full-shift PBZ samples	0.007–0.43	0.01(✕)	0.01(✕)	0.01(✕)	0.01(✕)	YES
Tapered steel roller bearing manufacturing, 2006 [151]	Metalworking fluid: Full-shift PBZ samples (thoracic particulates)	0.22–5	NR	NR	0.40(✕)	NR	YES
	Formaldehyde: Full-shift PBZ samples	ND–0.06	2(✓)	0.75(✓)	0.016(✓)	2(✓)	NO
Microwave popcorn plant, 2006 [153]	Diacetyl: Full-shift PBZ samples	ND–97.9	NR	NR	0.0005(✕)	0.02(✕)	YES
Polystyrene and foam manufacturing, 2006 [154]	Pentane: Full-shift PBZ samples	7–73	600(✓)	1000(✓)	120(✓)	600(✓)	NO
	Total dust: Full-shift PBZ samples	1.88	10(✓)	15(✓)	NE	10(✓)	NO
	Respirable dust: Full-shift PBZ samples	0.09	5(✓)	5(✓)	5(✓)	4(✓)	NO
Flock manufacturing facility, 2006 [155]	Respirable dust: Full-shift PBZ samples	0.01–0.60	5(✓)	5(✓)	5(✓)	4(✓)	NO
Cultured marble vanities, bath tubs, and shower walls and floors manufacturing, 2006 [157]	Total particulate: Full-shift PBZ samples	0.6–43	10(✕)	15(✕)	NE	10(✕)	YES
	Respirable particulate: Full-shift PBZ samples	0.09–0.40	5(✓)	5(✓)	5(✓)	4(✓)	NO
	Styrene: Personal breathing zone air samples	0.2–31	100(✓)	100(✓)	50(✓)	100(✓)	NO
	α-Methyl styrene: Full-shift PBZ samples	ND–0.6	100(✓)	100(✓)	50(✓)	100(✓)	NO
	Methyl methacrylate: Full-shift PBZ samples	0.1–2.8	100(✓)	100(✓)	100(✓)	50(✓)	NO
	Noise: Personal noise exposure doses	73.4–96.4 ^E^ 89.3–112.3 ^F^	85(✕)	85(✕) 90(✕)	85(✕)	80(✕) 87(✕)	YES
Aircraft fuel cells manufacturing, 2006 [158]	MEK: Full-shift PBZ samples	0.3–144	200(✓)	200(✓)	200(✓)	200(✓)	NO
	Acetone: Full-shift PBZ samples	0.3–145.6	750(✓)	1000(✓)	250(✓)	500(✓)	NO
	Toluene: Full-shift PBZ samples	0.05–6	50(✓)	200(✓)	100(✓)	50(✓)	NO
Poultry processing facility, 2006 [159]	Trichloramines: Full-shift PBZ samples	ND–0.000023	NR	NR	NE	NR	UNCLEAR
	Soluble chlorine: Full-shift PBZ samples	ND–0.0001	0.5(✓)	1(✓)	0.5(✓)	0.5(✓)	NO
Glass container manufacturer, 2005 [161]	Tin: Full-shift PBZ samples	ND–4.6	2(✕)	2(✕)	2(✕)	2(✕)	YES
	Monobutyltin trichloride: Full-shift PBZ samples	ND–1.5	NR	NR	NE	NR	UNCLEAR
	Hydrochloric acid: Full-shift PBZ samples	ND–0.17	5(✓)	7(✓)	7(✓)	2(✓)	NO
Computer services, 2005 [162]	Trichloroethylene: Full-shift PBZ samples	0.01–0.89	100(✓)	100(✓)	25(✓)	100(✓)	NO
	Trimethylbenzene: Full-shift PBZ samples	0.32–1.6	25(✓)	NR	25(✓)	25(✓)	NO
	2-butoxyethanol: Full-shift PBZ samples	4.2–9.3	NR	50(✓)	5(✓)	25(✓)	NO
Fabricated metal product manufacturing, 2005 [163]	Total particulates: Full-shift PBZ samples	0.1–7.6	10(✓)	15(✓)	NE	10(✓)	NO
	Copper in total dust: Full-shift PBZ samples	ND–0.087	1(✓)	1(✓)	1(✓)	1(✓)	NO
	Iron in total dust: Full-shift PBZ samples	0.04–4	5(✓)	10(✓)	5(✓)	5(✓)	NO
PTFE, thermoplastic rotating seals, subassembly systems and plastic mating component manufacturing, 2005 [164]	Airborne fiberglass: Full-shift PBZ samples	1.9–3.9 ^	NR	15(✓)	3^(✕)	NR	YES
Portland cement company, 2005 [165]	Total particulates: Full-shift PBZ samples	0.57–59.69	10(✕)	15(✕)	NE	10(✕)	YES
	Respirable dust: Full-shift PBZ samples	ND–0.96	5(✓)	5(✓)	5(✓)	4(✓)	NO
	Aluminium in total dust: Full-shift PBZ samples	0.02–0.92	10(✓)	15(✓)	5(✓)	10(✓)	NO
	Calcium in total dust: Full-shift PBZ samples	0.08–15.41	10(✕)	15(✕)	5(✕)	10(✕)	YES
	Iron in total dust: Full-shift PBZ samples	0.02–0.96	5(✓)	10(✓)	5(✓)	5(✓)	NO
	Magnesium in total dust: Full-shift PBZ samples	ND–0.56	10(✓)	15(✓)	NE	10(✓)	NO
Hardware (zinc casting department), 2005 [167]	Xylene: Full-shift PBZ samples	0.038–0.080	100(✓)	100(✓)	100(✓)	50(✓)	NO
	Ethyl benzene: Full-shift PBZ samples	0.0013–0.015	100(✓)	100(✓)	100(✓)	100(✓)	NO
	n-Butyl acetate: Full-shift PBZ samples	0.0057–0.52	150(✓)	150(✓)	150(✓)	150(✓)	NO
	Trimethylbenzene: Full-shift PBZ samples	0.004–0.4	25(✓)	NR	25(✓)	25(✓)	NO
	Diacetone alcohol: Full-shift PBZ samples	ND–0.20	50(✓)	50(✓)	50(✓)	50(✓)	NO
	Propylene glycol monoethyl ether acetate: Full-shift PBZ samples	0.003–0.42	NR	NR	NE	NR	UNCLEAR
Magnesium ingot, magnesium recycling and chemical by-products supplier and manufacturer, 2005 [169]	Carbon tetrachloride: Full-shift PBZ samples	ND–0.18	2(✓)	10(✓)	2(✓)	1(✓)	NO
	Hexachlorobenzene: Full-shift PBZ samples	ND–0.0069	NR	NR	NE	NR	UNCLEAR
Asphalt plant 1, 2005 [170]	Total particulate (diesel particulate): Full-shift PBZ samples	0.21–8.48	NR	NR	NE	NR	UNCLEAR
	Benzene-soluble fraction: Full-shift PBZ samples	ND–0.08	NR	NR	NE	NR	UNCLEAR
	Organic carbon: Full-shift PBZ samples	0.000064	NR	NR	NR	NR	UNCLEAR
	Elemental carbon: Full-shift PBZ samples	0.000005	NR	NR	NR	NR	UNCLEAR
Heavy metal fabrication operation, 2005 [171]	HDI monomer: Full-shift PBZ samples	0.000001–0.000004	0.2(✓)	NR	0.005(✓)	0.02(✓)	NO
	NCO monomer: Full-shift PBZ samples	0.3–1.9	0.02(✕)	NR	0.005(✕)	0.02(✕)	YES
	NCO oligomer: Full-shift PBZ samples	0.8–298	0.02(✕)	NR	0.005(✕)	0.02(✕)	YES
Microwave popcorn plant, 2004 [173]	Diacetyl: Full-shift PBZ samples	ND–0.004	NR	NR	NE	0.02(✓)	NO
	Total dust: Full-shift PBZ samples	0.02–0.3	10(✓)	15(✓)	NE	10(✓)	NO
	Respirable dust: Full-shift PBZ samples	0.01–0.06	5(✓)	5(✓)	5(✓)	4(✓)	NO
Microwave popcorn production, 2004 [174]	Diacetyl: Full-shift PBZ samples	ND–1.97	NR	NR	0.005(✕)	0.02(✕)	YES
	Acetoin: Full-shift PBZ samples	ND–1.82	NR	NR	NE	NR	UNCLEAR
Corrosive-resistant stainless steel and piping system fabrication facility, 2004 [175]	Nickel: Full-shift PBZ samples	0.032–0.156	0.5(✓)	1(✓)	0.015(✕)	0.5(✓)	YES
	Chromium: Full-shift PBZ samples	0.072–0.36	0.5(✓)	1(✓)	0.5(✓)	0.5(✓)	NO
	Manganese: Full-shift PBZ samples	0.01–0.34	5(✓)	5(✓)	1(✓)	0.05(✓)	NO
	Hexavalent Chromium: Full-shift PBZ samples	0.005–0.02	0.05(✓)	0.1(✓)	0.005(✓)	0.05(✓)	NO
Metal parts manufacturing, 2004 [176]	Respirable dust (particles not otherwise regulated): Full-shift PBZ samples	ND–5.9	5(✕)	5(✕)	5(✕)	4(✕)	YES
	Aluminium: Full-shift PBZ samples	0.003–0.98	10(✓)	15(✓)	5(✓)	10(✓)	NO
	Titanium: Full-shift PBZ samples	0.0067–0.19	10(✓)	15(✓)	LFL	10(✓)	NO
	Yttrium: Full-shift PBZ samples	ND–1.14	1(✕)	1(✕)	1(✕)	1(✕)	YES
	Vanadium pentoxide: Full-shift PBZ samples	0.00042–0.022	0.5(✓)	0.5(✓)	0.05(✓)	0.05(✓)	NO
Polyethylene and polypropylene plastics complex, 2004 [177]	Hexavalent chromium: Full-shift PBZ samples	ND–0.39	0.05(✕)	0.1(✕)	0.005(✕)	0.05(✕)	YES
Agri-business enterprise (potato processor), 2004 [180]	Noise: Personal noise levels	41–87.8 ^E^ 75.9–91.6 ^F^	85(✕)	85(✕) 90(✕)	85(✕)	80(✕) 87(✕)	YES
	Noise: Area noise levels	80–105 ^E^	85(✕)	85(✕) 90(✕)	85(✕)	80(✕) 87(✕)	YES
Microwave popcorn plant, 2003 [182]	Diacetyl: Full-shift PBZ samples	0.01–1.14	NR	NR	0.005(✕)	0.02(✕)	YES
	Acetoin: Full-shift PBZ samples	0.01–1.05	NR	NR	NE	NR	UNCLEAR
Foam cushion manufacturer, 2003 [183]	1-bromopropane: Full-shift PBZ samples	7–281	NR	NR	NE	NR	UNCLEAR
	2-bromopropane: Full-shift PBZ samples	0.08–0.68	NR	NR	NE	NR	UNCLEAR
Specialty chemical manufacturer, 2003 [184]	3-Amino-5mercapto-1,2,4-triazole: Full-shift PBZ samples	0.005–5.6	NR	NR	NE	NR	UNCLEAR
	Flumetsulam: Full-shift PBZ samples	0.0007–5.8	NR	NR	NE	NR	UNCLEAR
Custom concrete counter tops manufacturer, 2003 [185]	Noise: Dosimetry	74.6–84.2 ^E^	85(✕)	85(✕) 90(✕)	85(✕)	80(✕) 87(✕)	YES
	Respirable dust: Full-shift PBZ samples	0.8–10	5(✕)	5(✕)	5(✕)	4(✕)	YES
Aluminium oil cooler producer, 2003 [186]	Aluminium: Full-shift PBZ samples	0.017–0.25	10(✓)	15(✓)	5(✓)	10(✓)	NO
	Total particulate: Full-shift PBZ samples	0.11–1.3	10(✓)	15(✓)	NE	10(✓)	NO
	Trichloroethylene: Full-shift PBZ samples	7.1–7.6	100(✓)	100(✓)	25(✓)	100(✓)	NO
Turkey processing facility, 2003 [187]	Soluble chlorine: Full-shift PBZ samples	0.0000035–0.0000013	0.5(✓)	1(✓)	0.5(✓)	0.5(✓)	NO
	Trichloramine: Full-shift PBZ samples	ND–0.00016	NR	NR	NE	NR	UNCLEAR
Flexographic printing operation, 2003 [188]	Dimethylaminoethanol: Full-shift PBZ samples	0.02–5	NR	NR	NE	NR	UNCLEAR
	Dimethylisopropanolamine: Full-shift PBZ samples	0.04–2.9	NR	NR	NE	NR	UNCLEAR
Microwave popcorn plant, 2003 [189]	Diacetyl: Full-shift PBZ samples	0.06–0.64	NR	NR	0.005(✕)	0.02(✕)	YES
	Acetoin: Full-shift PBZ samples	ND–0.501	NR	NR	NE	NR	UNCLEAR
Metal valves and steam traps manufacturer, 2003 [190]	Toluene: Full-shift PBZ samples	0.20–0.34	50(✓)	200(✓)	100(✓)	50(✓)	NO
	Butyl acetate: Full-shift PBZ samples	0.34–0.57	150(✓)	150(✓)	150(✓)	150(✓)	NO
	Propylene glycol monoethyl ether acetate: Full-shift PBZ samples	0.36–0.58	NR	NR	NR	NR	UNCLEAR
	Cyclohexanone: Full-shift PBZ samples	0.59–1	25(✓)	50(✓)	25(✓)	10(✓)	NO
	Decane: Full-shift PBZ samples	0.32–0.37	NR	NR	0.5(✓)	NR	NO
	Methyl ethyl ketone: Full-shift PBZ samples	2.3–4.6	200(✓)	200(✓)	200(✓)	200(✓)	NO
Metal phosphide-based fumigant manufacturer, 2003 [191]	Total dust (particulates not otherwise regulated): Full-shift PBZ samples	0.047–0.18	10(✓)	15(✓)	NE	10(✓)	NO
	Aluminium: Full-shift PBZ samples	0.01–0.02	10(✓)	15(✓)	5(✓)	10(✓)	NO
	Nickel: Full-shift PBZ samples	ND–0.001	0.5(✓)	1(✓)	0.015(✓)	0.5(✓)	NO
	Titanium: Full-shift PBZ samples	ND–0.001	10(✓)	15(✓)	NE	10(✓)	NO
	Lithium: Personal breathing zone samples	ND–0.001	0.025(✓)	0.025(✓)	0.025(✓)	0.02(✓)	NO
Flexible packaging and pressure sensitive material manufacturer, 2003 [192]	Formaldehyde: Full-shift PBZ samples	0.04–0.09	2(✓)	0.75(✓)	0.016(✕)	2(✓)	YES
	Acetaldehyde: Full-shift PBZ samples	0.02–0.06	100(✓)	200(✓)	LFC	20(✓)	NO
Microwave popcorn manufacturer, 2003 [194]	Diacetyl: Full-shift PBZ samples	ND–18	NR	NR	0.005(✕)	0.02(✕)	YES
	Acetoin: Full-shift PBZ samples	ND–0.07	NR	NR	NE	NR	UNCLEAR
Valve manufacturing, 2002 [195]	Phenol: Full-shift PBZ samples	ND–0.08	5(✓)	5(✓)	5(✓)	2(✓)	NO
	Ammonia: Full-shift PBZ samples	ND–3.7	25(✓)	50(✓)	25(✓)	25(✓)	NO
	White spirits: Full-shift PBZ samples	1.71–5.41	100(✓)	500(✓)	350(✓)	NR	NO
	Cumene: Full-shift PBZ samples	ND–0.09	25(✓)	50(✓)	50(✓)	25(✓)	NO
	Toluene: Full-shift PBZ samples	0.02–0.13	50(✓)	200(✓)	100(✓)	50(✓)	NO
	Trimethylbenzene Full-shift PBZ samples	0.12–2.2	25(✓)	NR	25(✓)	25(✓)	NO
Titanium and aluminium commercial airplane parts manufacturer, 2002 [196]	Metalworking fluid: Full-shift PBZ samples	ND–1.84	NR	NR	0.4(✕)	NR	YES
Electroplated strip steel manufacturer, 2002 [197]	Copper: Full-shift PBZ samples	0.0002–0.04	1(✓)	1(✓)	1(✓)	1(✓)	NO
	Iron: Full-shift PBZ samples	0.0009–0.004	5(✓)	10(✓)	5(✓)	5(✓)	NO
	Nickel: Full-shift PBZ samples	0.0008–0.1	0.5(✓)	1(✓)	0.015(✕)	0.5(✓)	YES
	Zinc: Full-shift PBZ samples	0.0004–0.02	5(✓)	5(✓)	5(✓)	NR	NO
	2.6-di-tert-butyl-p-cresol (butylated hydroxytoluene): Full-shift PBZ samples	0.001–0.004	NR	NR	10(✓)	NR	NO
Rubber moulded parts, rubber to metal mould bonded bushings, Teflon lined bonded bushings, and rubber compounds manufacturer, 2002 [198]	Total particulate: Full-shift PBZ samples	0.04–1.71	10(✓)	15(✓)	NE	10(✓)	NO
	Respirable particulate: Full-shift PBZ samples	0.17	5(✓)	5(✓)	5(✓)	4(✓)	NO
Air compressor manufacturer, 2002 [199]	Total or thoracic metalworking fluid: 8-hour TWA PBZ samples	0.10–1.98	NR	NR	0.4(✕)	NR	YES
	Total or thoracic extractable metalworking fluid: 8-hour TWA PBZ samples	ND–1.16	NR	NR	NE	NR	UNCLEAR
	n-Butyl acetate: Full-shift PBZ samples	0.08–1.8	150(✓)	150(✓)	150(✓)	150(✓)	NO
	MIBK: Full-shift PBZ samples	0.08–2.2	50(✓)	100(✓)	50(✓)	50(✓)	NO
	Xylene: Full-shift PBZ samples	0.19–3.1	100(✓)	100(✓)	100(✓)	50(✓)	NO
Sofa cushion manufacturer, 2002 [200]	1-Bromopropane: Full-shift PBZ samples	6.3–143	NR	NR	NE	NR	UNCLEAR
	2-Bromopropane: Full-shift PBZ samples	0.1–1.4	NR	NR	NE	NR	UNCLEAR
Neon tube manufacturing, 2002 [201]	Mercury: Full-shift PBZ samples	0.03	0.05(✓)	0.1(✓)	0.05(✓)	0.02(✕)	YES
Flexographic printing operation, 2002 [202]	Dimethylaminoethanol: Full-shift PBZ samples	0.18–5.16	NR	NR	NE	NR	UNCLEAR
	Dimethylisopropanolamine: Full-shift PBZ samples	0.66–17.08	NR	NR	NE	NR	UNCLEAR
Glass funnel and panel manufacturer, 2002 [203]	Heat stress: Area WBGT measurements	32.7–39.3	30 ^G^(✕)	NR	26.7 ^G^(✕)	Varies	YES
Automotive brake calipers and drum manufacturer, 2002 [204]	Metalworking fluid aerosol: Full-shift PBZ samples	ND–0.41	NR	NR	0.4(✕)	NR	YES
	Thoracic particulates: Full-shift PBZ samples	0.14–0.69	NR	NR	NE	NR	UNCLEAR
Seat cushion manufacturer, 2002 [206]	1-Bromopropane: Full-shift PBZ samples	60–381.2	NR	NR	NE	NR	UNCLEAR
	2-Bromopropane: Full-shift PBZ samples	0.01–0.55	NR	NR	NE	NR	UNCLEAR
Specialty, nonferrous metal-alloy billet producer, 2001 [207]	Hexavalent chromium: Full-shift PBZ samples	ND–0.00000038	0.05(✓)	0.1(✓)	0.005(✓)	0.05(✓)	NO
	Cobalt: Full-shift PBZ samples	ND–0.000276	0.1(✓)	0.1(✓)	0.05(✓)	0.1(✓)	NO
	Niobium: Full-shift PBZ samples	ND–0.00001	NR	NR	NE	NR	UNCLEAR
	Nickel: Full-shift PBZ samples	ND–1.373	0.5(✕)	1(✕)	0.015(✕)	0.5(✕)	YES
Potato products manufacturer, 2001 [208]	Total particulate: Full-shift PBZ samples	0.038–0.527	10(✓)	15(✓)	NE	10(✓)	NO
Catalyst manufacturer, 2001 [209]	Nickel: Full-shift PBZ samples	0.005–16.15	0.5(✕)	1(✕)	0.015(✕)	0.5(✕)	YES
Wire rope products manufacturer, 2001 [210]	Asphalt fume (total particulate): Full-shift PBZ samples	0.6–3.2	5(✓)	NR	5(✓)	5(✓)	NO
	Asphalt fume–benzene-soluble fraction: Full-shift PBZ samples	0.2–1.2	5(✓)	NR	NR	5(✓)	NO
	Noise: Personal noise dosimetry	83–103.2 ^E^ 89.6–105.5 ^F^	85(✕)	85(✕) 90(✕)	85(✕)	80(✕) 87(✕)	YES
Instrumentation and component manufacturer, 2001 [211]	1-bromopropane: Full-shift PBZ samples	0.02–0.63	NR	NR	NE	NR	NO
	2-bromopropane: Full-shift PBZ samples	ND	NR	NR	NE	NR	NO
Woodworking operation (Garage interior component production), 2001 [212]	Total wood dust particulates: Full-shift PBZ samples	0.39–2.6	NR	15(✓)	1(✕)	NR	YES
	Respirable wood dust particulates: Full-shift PBZ samples	0.028–1.9	NR	5(✓)	1(✕)	NR	YES
Shear, scissors and thread manufacturer, 2001 [213]	Total or thoracic metalworking fluid: 8-hour PBZ samples	0.78–3.95	NR	NR	0.4(✕)	NR	YES
	Total or thoracic extractable metalworking fluid: 8-hour PBZ samples	0.66–3.78	NR	NR	NE	NR	UNCLEAR
Nonwoven and specialty fibres manufacturer, 2001 [214]	Total dust: Full-shift PBZ samples	0.033–0.099	10(✓)	15(✓)	NE	10(✓)	NO
	Fibres: Full-shift PBZ samples	0.008–0.022	NR	15(✓)	3^(✓)	NR	NO
	Sulphuric acid mist: Personal breathing zone samples	ND–0.087	1(✓)	1(✓)	1(✓)	0.05(✕)	NO
Portland cement company, 2001 [215]	Total dust: Full-shift PBZ samples	0.127–3.80	10(✓)	15(✓)	NE	10(✓)	NO
Aircraft support centre, 2001 [216]	Total dust (particulate not otherwise classified): Full-shift PBZ samples	0.09–0.34	10(✓)	15(✓)	NE	10(✓)	NO
	Dipropylene glycol butyl ether: Full-shift PBZ samples	0.056–0.40	NR	NR	NR	NR	UNCLEAR
	Tripropylene glycol methyl ether: Full-shift PBZ samples	0.13–0.67	NR	NR	NR	NR	UNCLEAR
Electrical parts, starters/generators, generator control units, fans, hydraulics, wheels, and breaks assembly shops, 2001 [217]	Toluene: Full-shift PBZ samples	1.09–2.07	50(✓)	200(✓)	100(✓)	50(✓)	NO
	HDI-based polyisocyanate: Full-shift PBZ samples	ND–1.56	0.2(✕)	NR	0.005(✕)	0.02(✕)	YES
Microwave popcorn production, 2001 [218]	Diacetyl: Full-shift PBZ samples	0.19–86.9	NR	NR	0.005(✕)	0.02(✕)	YES
	Acetoin: Full-shift PBZ samples	0.05–11.7	NR	NR	NE	NR	UNCLEAR
Flock production, 2000 [219]	Respirable dust: Full-shift PBZ samples	0.02–0.08	5(✓)	5(✓)	5(✓)	4(✓)	NO
	Fibre dust: Full-shift PBZ samples	0.1–0.2 ^	NR	15(✓)	3^(✓)	NR	NO
Flat, clear glass producer, 2000 [221]	Respirable dust: Full-shift PBZ samples	0.31–4.86	5(✓)	5(✓)	5(✓)	4(✕)	YES
	Crystalline silica dust: Full-shift PBZ samples	0.09–0.35	0.04(✕)	0.05(✕)	0.05(✕)	0.1(✕)	YES
	Total dust: Full-shift PBZ samples	0.25–0.85	10(✓)	15(✓)	NE	10(✓)	NO
	Total dust (adipic acid concentration): Full-shift PBZ samples	0.02–0.14	NR	NR	NE	NR	UNCLEAR
Automotive foam cushion manufacturing, 2000 [222]	2,4-TDI: Full-shift PBZ samples	ND–0.000004	0.2(✓)	0.02(✓)	LFL(✓)	0.02(✓)	NO
	2,6-TDI: Full-shift PBZ samples	ND–0.000004	0.2(✓)	0.02(✓)	LFL(✓)	0.02(✓)	NO
Flocking facility, 2000 [223]	Respirable dust: Full-shift PBZ samples	0.04–0.062	5(✓)	5(✓)	5(✓)	4(✓)	YES
	Respirable fibres: Full-shift PBZ samples	0.04–0.11	NR	15(✓)	3^(✓)	NR	NO
Aircraft engine facility, 2000 [224]	4,4-methylenedianiline: Full-shift PBZ samples	ND–0.00042	0.1(✓)	0.010(✓)	LFL(✓)	0.01(✓)	NO
	Methanol: Full-shift PBZ samples	3.6–22	200(✓)	200(✓)	200(✓)	200(✓)	NO
Military aircraft manufacturer, 2000 [225]	4,4-methylenedianiline: Full-shift PBZ samples	ND–0.001364	0.1(✓)	0.010(✓)	LFL(✓)	0.01(✓)	NO
	MDI-based polyisocyanate: Full-shift PBZ samples	ND–0.00108	0.2(✓)	0.02(✓)	0.005(✓)	0.02(✓)	NO
	HDI: Full-shift PBZ samples	0.0000014–0.0000019	0.2(✓)	NR	0.005(✓)	0.02(✓)	NO
	HDI-based polyisocyanate: Full-shift PBZ samples	ND–0.0002	0.2(✓)	NR	0.005(✓)	0.02(✓)	NO
Backhoe, crawler dozers and rough terrain forklifts manufacturer, 2000 [226]	Aluminium metal: Full-shift PBZ samples	ND–0.013	10(✓)	15(✓)	5(✓)	10(✓)	NO
	Iron metal: Full-shift PBZ samples	0.06–6.8	5(✕)	10(✓)	5(✕)	5(✕)	YES
	Manganese metal: Full-shift PBZ samples	0.02–0.81	5(✓)	5(✓)	1(✓)	0.2(✕)	YES
	Nickel metal: Full-shift PBZ samples	ND–0.004	0.5(✓)	1(✓)	0.015(✓)	0.1(✓)	NO
	Total or thoracic metalworking fluid: Full-shift PBZ samples	ND–7.92	NR	NR	0.4(✕)	NR	YES
	Total or thoracic extractable metalworking fluid: Full-shift PBZ samples	ND–1.03	NR	NR	NE	NR	UNCLEAR
Automobile transmission plant, 2000 [228]	Total or thoracic metalworking fluid particulate: Full-shift PBZ samples	0.12–0.51	NR	NR	0.4(✕)	NR	YES
	Total or thoracic particulate: Full-shift PBZ samples	0.04–0.74	NR	NR	NE	NR	UNCLEAR
Aircraft support centre, 2000 [229]	Particulates respirable fraction: Full-shift PBZ samples	0.05–0.59	5(✓)	5(✓)	5(✓)	4(✓)	NO
	Particulates inhalable fraction: Full-shift PBZ samples	0.13–4.01	10(✓)	15(✓)	NE	10(✓)	NO
	Iron inhalable fraction: Full-shift PBZ samples	ND–0.158	5(✓)	10(✓)	5(✓)	5(✓)	NO
Precious metal recycling facility, 2000 [231]	Silver: Full-shift PBZ samples	0.14	0.01(✕)	0.01(✕)	0.01(✕)	0.01(✕)	YES
Hydraulic commercial and industrial elevator production, 2000 [233]	Total welding fume: Full-shift PBZ samples	5.44–6.1	NR	NR	5(✕)	NR	YES
	Manganese fume: Full-shift PBZ samples	0.23–0.31	1(✓)	1(✓)	1(✓)	0.2(✕)	YES
Wire harness and heating, ventilation, and air conditioning components assembly shop, 2000 [234]	Lead: Full-shift PBZ samples	ND–0.000004	0.15(✓)	0.050(✓)	0.050(✓)	0.15(✓)	NO
	Tin: Full-shift PBZ samples	ND–0.55	0.1(✕)	0.002(✕)	0.002(✕)	0.1(✕)	YES
	Noise: noise dosimetry results	78.9–90.2 ^F^	85(✕)	85(✕) 90(✕)	85(✕)	80(✕) 87(✕)	YES

^A^ Exposure limit for noise in dBA unless indicated otherwise; milligram per cubic meter (mg/m^3^) for particulates; parts per million (ppm) for solvents vapours and gases, and meters per second squared for vibration | ^B^ Not regulated | ^C^ Non detect | ^D^ Not established| ✓ Complies with health and safety standard| ✕ Does not comply with health and safety standard| ^E^ Values derived using OSHA instrument settings| ^F^ Values derived using NIOSH instrument settings | ^G^ Limit is for acclimatised, healthy, physically fit men engaged in moderate continuous physical activity, ^H^ Lowest feasible level.

## 4. Discussion

Table 1 and Table 2 depicts evidentiary and interpretive proof of worker, employer, government and labour union concern relating to workplace exposure, manifested in formal requests for investigations. The initiators of the exposure investigations covered in this review paper emanated from stakeholders from an array of sub-industries within the manufacturing sector as well as government, and related to both chemical and physical hazard types. The volume of investigations triggered by complaints seem minimal to moderate, a view also shared by Spieler [6]. This compared to the number of establishments within the manufacturing sector totalling some 358,000, as well as the yearly occupational disease (OD) cases ranging between 258,000 and 329,000, reported between 2015 and 2019 within the U.S. manufacturing sector alone [236,237]. These statistics are suggestive of muted concerns of exposure as well as insufficient risk perception by some stakeholders within the sector. The authors of this review paper however submit that the relevant institutions are proactively attending to received exposure concerns in an efficient manner, under persistent staffing challenges.

The moderate volume of requested investigations may also be an acceptance of a declining influence of governmental institutions in OHS matters [238]. Administratively, the moderate number of complaints are so in part, due to the screening process leading to their dismissal or withdrawal on grounds of lack of cooperation and jurisdiction and late filing [6]. Smith [239] also argued that the small volume of worker complaints directed for investigation are also in part to the alleged perception of resource consumption with minimal impact.

In support of worker-initiated requests for investigations, Smith [239] expounds that workers should be encouraged to continuously lodge complaints as they uncover other OHS violations during investigations. Due to the legal responsibilisation of worker duties, workers therefore have an unquestionable moral duty of protecting themselves against risks and hazards by voicing concerns as they arise [240]. Risk perception however, plays an important role on how workers perceive and manage these risks and hazards [241]. Risk perception itself is predicted using models, such as psychometric models and cultural theory of risk perception, with low correlation to worker perception [241]. Given the risk associated with hazard exposure in an occupational setting, it is thus comprehensible that the working conditions encountered at the workplace should be of great concern for workers and other stakeholders [242]. Migrant workers, blue-collar workers, samplers, production workers, machinists, and lower level supervisory personnel continue to be the most highly exposed job categories to identified occupational health hazards [236,243,244]. With regard to risk perception related to noise, employees in workplaces with excessive noise levels have high risk perception compared to those in workplaces with noise exposure levels around the exposure limits, reported Bockstael, De Bruyne [245].

From a global point of view, there is a need for the continual up keeping of occupational hygiene exposure data sets for some of the identified hazards as well as better OHS regulatory policies. In the case of exposure data sets, these become valuable during occupational exposure assessment initiatives and indicates that workers are indeed exposed [246]. With regard to the nagging concern of noise exposure as an example, noise data bases such as that available from the OSHA-administered Integrated Management Information System [247] and the NIOSH noise measurement database [248], can prove useful during prioritisation of targeted exposure interventions. This still remains relevant today as noise exposure and hearing loss are still contemporary within the cycles of occupational hygienists who are required to identify, evaluate and control noise; whilst employers are expected to provide resources for control; whereas policymakers, the other important stakeholders, have the mandatory responsibilities to regulate exposure [249]. With regard to specific OHS laws related to noise, the measured noise levels above the exposure limits indicated in Table 2 highlights their weaknesses. As an example, current noise regulations, worldwide, and in general, allow for hearing protection device (HPD) use as a default control within hearing conservation programs which is proving to be problematic as some 34% noise-exposed workers from the U.S. have reported non-use thereof. Instead of using HPDs as a short-term control measure, workplaces have tended to neglect the implementation of feasible engineering noise controls which reduces noise at the source [250]. Untreated noise implies that resultant exposure will remain prevalent well into the future.

This therefore implies that companies should thus attach great value in collecting exposure data as part of demonstrating legal compliance, for instituting and checking the efficacy of implemented preventive measures [251]. Regrettably, in the U.S., large corporations have tended to reduce workplace exposure sources commensurate with historical changes in regulated exposure limits [252], than in response to worker concerns. On the other hand, no publicly available occupational exposure measurements are available in SA, though companies are required by OHS laws to report exposure data to regulatory authorities.

### 4.1. Concern and Perception of Hazards and Risks by Workers

Workers’ risk perception leading to formal complaints to responsible government institutions relating to occupational health hazard exposure is shown in Table 1 and Table 2. In formalising the complaints, workers merely exercised their legal rights afforded within OHS laws [1,2,3]. Although workers may know the unacceptability of unsafe working conditions, they often lack knowledge on whom to consult to remedy infractions, consequently leading to acceptance of unsafe conditions as part of daily operations [253]. To highlight the importance of correct risk perception, Robinson and Smallman [254] posit that workplaces whose workers are encouraged and empowered to actively influence OHS tend to conduct work in a safe and healthier manner.

Workers perceive and interpret hazards differently based on gender, duration of employment and prevailing safety climate. A hazard may be perceived as trivial and hidden by one worker, whilst the same hazard is perceived as obvious and emerging by another [255,256]. With regard to gender differences, men have lower risk perceptions to health hazards compared to woman as a result of risk familiarity [257,258,259]. Whereas, the high risk perception in women is linked to their social roles of being nurtures and care providers which is generally related to health and safety issues [259]. Men consequently have high OD burden due to their low risk perception notwithstanding the skewed employment demographics within the manufacturing sector. Leoni [260] also reported that risk perception correlates are higher in single parents, elderly workers and workers with completed tertiary education. Given this view and the uncertainty associated with this perception, the relationship between gender and risk perception still requires further studies [259].

The evident disparity in risk perception also extends to new workers into a job compared to experienced workers in the same job [242,255,260,261]. To increase the success of workplace health interventions, Robinson, and Smallman [262] suggested that employers and regulatory authorities should raise the health and safety awareness levels of new entrants and younger workers. Employment status, such as contract work, economic, and remuneration factors, and inadequate regulatory controls, are also identified as playing important roles in worker risk perception [263].

Arezes and Miguel [264], Fleming, Flin [265], and Garcia, Boix [261] reported in their respective studies that prevailing safety climate within an enterprise greatly influences workers’ behaviour towards identified hazards whilst at work. Enterprise factors such as extended shift cycles, employees’ characteristics, attitude and job requirements are also contributory to worker risk perception [266]. Individual risk perception and the value of self-preservation is also an outcome of safety climate playing an indirect; yet predictive role in the use of protective equipment [264]. In the same breath, Frenkel, Priest [242], in their study reporting worker perception to occupational health and safety, reported that majority of workers in their sample were able to identify one or more occupational health hazards, such as noise, extremes of indoor temperature, fumes, dust and dangerous chemicals in their workplaces. This highlighting an enterprise with an effective risk management strategy was found to be intriguing in that it empowered all workers to correctly identify hazards in an aligned manner [255]. Despite this noted success, many workers however still lack in skills of identifying effective hazard preventive strategies which compounds the problem [267]. There is also a credible claim that the workers’ perception to exposure and risk is largely influenced by disease latency, as workers tend to be more concerned about exposure giving rise to immediate, medium-term effects compared to chronic health effects such as cancer and noise-induced hearing loss (NIHL) [257]. Of the identified health hazards in this review, noise is by far the most perceived occupational health hazard by workers as a danger to their health [257,258], this in spite of market availability of effective noise control measures and workplace noise regulations [257]. Frenkel, Priest [242], however, contended that newer employees into a job do not appear to perceive noise exposure as a health hazard indicating underestimation of prevailing risks. There however remains no definite and contemporary literature indicating that workers have accurate risk perceptions [265]. Therefore caution should be exercised when attempting to describe worker risk perception as segments of those workers with high risk perceptions are more likely predisposed to other health issues as burnout, anxiety, and depression; and are also the most dissatisfied with their jobs [268].

Although workers are empowered by health and safety laws to receive information related to workplace hazards [269], the information received is not always adequate. Workers’ right to know and to be informed about these hazards has also not entirely eliminated ODs from the workplace [270]. Rikhotso, Harmse [271] found that information provided to workers enrolled in a hearing conservation programme of a chemical manufacturing company was inadequate. Workers who are inadequately informed about health hazards have higher prevalence of ODs [242]. Contemporary health and safety laws assign workers greater responsibilities that make them accountable, liable, and sanctionable as opposed to the old system where they were seen as victims and offenders. This responsibilisation strategy can however cloud employer and worker health and safety responsibilities if not well understood [272].

The law also affords workers the right to refuse dangerous work, however, the action of refusing dangerous work will not in itself result in the improvement of workplace conditions [273]. The right to refuse dangerous work is reportedly the most exercised by union-affiliated workers [267]. Kerr [274] argued that workers are hindered in exercising their rights as they cannot force the employer to comply with health and safety laws as the enforcement duty has been legally placed on health and safety inspectors [273,274]. Undoubtedly, the guaranteeing of workers’ right to a hazard-free workplace continues to be an issue of central debate within the social justice context [275].

### 4.2. Employer Concerns and Perception of Hazards and Risks

Employers carry the unquestionable bulk of the responsibility of providing healthy and safe workplaces [275,276], and employ the services of trained specialists to fulfil and uphold this legal responsibility [276]. This ultimate employer responsibility stems from the fact they created the hazards and risks, and also decide how work is performed [276,277]. In that regard, workplaces with identified occupational health hazards erodes worker job satisfaction [242], erodes profit, reduces investment opportunities, as well as increases staff turnover and absenteeism [278]. In self-regulatory regimes, the identified hazards imply that employers have in large not fulfilled their legally imposed duty of providing hazard and risk free workplaces [279], notwithstanding the risk acceptability principles.

For the purpose of clarity regarding this matter, employer concern to occupational hazards and risks are also shown in Table 1 and Table 2. That employers initiated the highest number of exposure investigations compared to employees and unions has an unsurprising historical legal background. To this effect, initial OHS laws gave employers the ultimate responsibility of providing and maintaining the health of employees until recently, whereby employees are also charged with certain duties in so far as preserving health at work is concerned. From another point of view, employers also have a historic advantage of having institutional knowledge and better awareness with regard to health and safety arrangements required for legal compliance with OHS laws, prompting them to request exposure investigations [10]. Additionally, employers are also coerced into requesting exposure investigations by the natural deterrence accompanying pending regulatory inspections as demonstration of commitment to attaining legal compliance with OHS laws [280], and a shift in regulatory approaches incorporating OHS management systems as part of legal compliance [281].

Occupational hygienists in particular, play a proactive role in influencing risk perception by employers and subsequent establishment of occupational health programmes [276]. Bian and Keller [282] stated that employers’ risk perception is also influenced by a country’s culture. To improve the overall worker behaviour and attitude toward interventions intended for health and safety, visible management and commitment is required [261]. Concurrently, to show commitment to risk management, employers have included as a key governance theme the continuous identification, assessment and management of risks [283]. Nonetheless, employers are critiqued for not leveraging the participatory approach advocated by OHS laws by promoting the role of Health and Safety Representatives in the decision-making process, with the aim of encouraging the participation of all workers in workplace risk reduction efforts [284]. Where risks from hazards have resulted in adverse impact is indicative of a company’s failure to use their risk prevention knowledge [285]. Therefore, responsible and committed employers should stay abreast of advances in OHS science and new technologies intended for hazard assessment and control to improve their decision-making in the related field [276]. In that regard, to increase the risk perception of employers within enterprises, an introduction of a penal and reward system can be implemented to continuously improve risk reduction efforts [286].

### 4.3. Worker Representative Concerns and View on Risks and Hazards

Other legally recognised stakeholders with vested interest in occupational hazards include labour unions and Health and Safety Representatives, acting as worker voices [1,2,4]. In SA and the UK, relevant OHS legislation affords workers a reporting platform upon which issues such as exposure to occupational hazards can be progressively reported, inclusive of the Health and Safety Representative, Health and Safety Committee, employer and the inspector [2,4]. As shown in Table 1 and Table 2, U.S. labour union-initiated health hazard evaluations conducted by NIOSH also contributed a moderate share of exposure investigations to occupational hazards compared to those initiated by workers and employers, similar to employer and worker-initiated workplace exposure investigations. A contributory factor to the moderate union-initiated exposure investigations may be due to employers’ reported strategy of screening-out unionised and pro-union workers during job interviews, contends Beaumont and Townley [287]. Additionally, some workplaces remain non-unionised, thus minimising labour union involvement in initiating exposure investigations [254]. Workplace unionisation in itself, a consequence of protest by workers in response to prevailing occupational hazards at different workplaces, has been criticised for accompanying long work shifts and faster work pace, factors linked to an increase in ODs, argued Fairris [288]. According to Robinson and Smallman [254], union participation in workplace health and safety issues, however minimal, should be encouraged. From a legal view point, Segall [289] argued that unions are however not legally liable for failures in the control of occupational hazards as well as enforcing related health and safety standards. The control of occupational hazards lies with employers, whereas the enforcement of health and safety standards is a legal duty of regulatory inspectorates [289,290].

In so far as labour union involvement in workplace health and safety, Jacques [290] posited that labour unions are rightly involved as workers affected by ill health resulting from exposure to occupational hazards are from their membership. In that respect, labour unions deserve recognition for efforts made on health and safety matters. A case in point of labour union success in worker health and safety has been their instrumental role in the enactment of workplace health and safety legislation requiring institution of basic controls measures for prevailing occupational hazards, affirmed [290]. In response of failing OHS programmes, unions have proactively initiated alternative health and safety initiatives such as the Triangle of Prevention (TOP), as an example, allowing for an engagement between a union and company management to track the number of identified hazards as well as their mitigation [291]. Whilst not inclusive, McQuiston, Cable [291] asserted that TOP had the potential to strengthen the effectiveness of existing OHS programmes. Another union-initiated health and safety intervention with reported success included a worker training programme that enrolled union members. Post-training, a decline in OHS incidence metrics, credited to the success of the initiative, were noted [292].

In criticism directed towards labour unions, Brown [238] argued that health and safety concerns brought to the union’s attention by the rank and file members were not prioritised. Additionally, Robinson and Smallman [254] also argued that worker rights in regard participation in OHS initiatives to improve the health and safety at the workplace are still denied at some workplaces, regardless of union representation. More than ever, unions should attend to workplace realities experienced by workers and action such problems into demands for improving health and safety, argues Vogel [293]. In spite of prevailing active labour union involvement in OHS issues, conclusively, the success of worker health and safety strategies require joint cooperation between employers/workers and employers/labour unions. This stakeholder participation is a critical factor in regulatory endeavours of lowering ODs and injuries [254,294,295].

### 4.4. Concern of Exposure and Inadequacy of Workplace Exposure Limits

Table 2 also shows a generic comparison of measured exposure levels against exposure limits from SA, OSHA, HSE, and NIOSH. Comparison of the measured exposure levels shown in Table 2 enables occupational hygienists and associated professions to make risk-based decisions on the need for exposure mitigation [296]. In the absence of an international harmonisation on these limits, differences in compliance outcomes were noted in some instances for the same exposure value. In a case of chemical hazards, the extent of exposure is underestimated by both employers and workers due to lack of awareness of chemical names and their toxic effects [297]. In making determinations relating to compliance with health and safety standard regulated via the Occupational Safety and Health Act 1970, NIOSH is required to forward copies of outcomes to the Department of Labor (OSHA), regulatory inspectorate for enforcement action [21].

These mandatory exposure limits have been developed to protect workers and to provide for workplace regulation of the various occupational hazards [298,299,300]. These exposure limits create an equity relief whereby workers are entitled to work in a safe and healthy workplace without having to choose between health and safety or their jobs [273]. The stringency of exposure limits currently in use however reflects industry and labour interests [301]. Further, these exposure limits do not reflect individual workers’ preferences [302], whilst also not accounting for combined effects which may underestimate risks [303]. Consequently, employers only need to demonstrate that exposure levels are below the limits and thereafter are not mandated to do further risk control [303], to the detriment of workers. Therefore, to increase compliance, exposure limits should be sufficiently protective to workers and fair to employers, argued Vincent [300]. A further concern for Occupational hygienists is the exponential development of new chemicals, some highlighted in Table 2, which continue to outpace the establishment of exposure limits and air sampling and analytical methods [27].

## 5. Limitations

Due to differences in the OHS legislative arrangements and reporting methods between that from U.S., SA, and the UK, no comparisons could be deduced about aspects discussed in this paper. The discussions and conclusions made in this review paper are reflective of the state of OHS affairs in the U.S. on the relevant topics. The inference of the volume of conducted HHE investigations was solely based on numerical counts in the absence of records indicating the number of requested investigations over the covered period. The authors also acknowledge the intensive and time-consuming nature of conducting occupational hygiene measurements, investigations and actual reporting, which further complicates the issue. The statement on the justification of complaints was solely based on exposure data derived through occupational hygiene measurement techniques, and excluded biological exposure indices and questionnaire as complementary data collection tools which also used during these investigations, due to their inherent complexities. Additionally, statements on compliance to exposure limits considered the worst case scenario (highest quantified value) for each quantified occupational hazard. The discussion and conclusions made in this review paper are made against these methodological constraints.

## 6. Implications of Study Findings on the Role of Occupational Hygienists in Shaping Risk Perceptions

The role of OHS specialists, including occupational hygienists, remains little understood in industry. Occupational hygiene, as a specialised profession, is not limited to workplace monitoring and report writing, but is legally empowered to propose effective preventive measures for the advancement of worker health [304]. The effectiveness of OHS specialists such as occupational hygienists is currently questionable in view of the unacceptable leading OD metrics worldwide [305]. Occupational hygienists in particular, should reclaim and shape the professional discourse with regard to hazard and risk management processes in industry, which has a bearing on how occupational health hazards are perceived and eventually treated. In support of this view, occupational hygienists use scientific tools for characterising risks, which incorporate variability in exposure estimates and the dose-response curve scenarios [296]. In SA as an example, OHS regulations remain ambiguous with regard to specific OHS professions empowered to conduct inferred hazard identification and risk assessments [306,307,308]. This often creates legal and operational tension between OHS professionals stemming from divergences in descriptors used during risk ranking, consequence definitions, as well as impact rankings amongst others, when identified risks and hazards are being considered for treatment prioritization. Additionally, occupational hygienists are professionally trained and capable of recognizing and explaining mitigating factors and for determining true risks [27]. Due to their delayed health impacts in the main, occupational health hazards are often overlooked for treatment to occupational safety hazards. Conclusively, risk perception by different stakeholders is an important factor which should be considered amidst the ongoing unabated prevalence of ODs from industry.

## 7. Conclusions

The synthesised literature covering two decades from the year 2000 indicated that employers, workers, and unions, combined at 86%, were the main initiators of conducted exposure investigations. These stakeholders initiated the exposure investigations through the discharge of legal duties afforded by prevailing occupational health and safety laws, which promotes active participation in the management of issues of concern. The investigated exposure concerns related to both chemical and physical hazard types, for which exposure limits currently exist in the main, and all quantifiable through occupational hygiene techniques. The quantification of prevailing exposure levels to these occupational health hazards forms the basis for making judgements on the extent of workplace exposure and justification of exposure concerns. In spite of prevailing exposure limits and current legal arrangements of exposure investigations workers are still negatively impacted by workplace exposure from identified occupational health hazards. The implementation of preventive measures for protection against hazards is however influenced by risk perception and concern among workers, employers and national labour unions, reflected as complaints forwarded to relevant government institutions. Workers with a lack of concern to risks and hazards will not seek or pressure the employer to implement remedial actions, report the infraction to regulatory inspectorates for further investigation nor adequately use and follow provided protective measures. To promote a culture of prevention at the workplace, changes in behaviour and attitudes of workers towards hazards is needed [309]. Similarly, employers with inadequate risk perception to hazards will not be compelled to initiate risk reduction actions. In the case of workers, high risk perception is central to their cause and often leads to the detection and solution-seeking initiatives for identified health problems [310]. Workplace studies reporting noncompliance to hazards such as noise, thermal stresses, vibration, electromagnetic fields, ionizing radiation, chemical substances, and dusts should spring labour inspectorates into intensive enforcement activities [258]. Exposure investigations conducted by NIOSH in the U.S., as shown in Table 1 and Table 2, confirm the presence in the workplace, especially the manufacturing sector of a myriad of occupational health hazards. These investigations, initiated by various stakeholders, have had a positive effect in inducing workplaces to abate exposure. However, abatement efforts with regard to noise in cited literature leans towards implementation of fulltime hearing conservation programs to engineering noise controls. This implies that noise exposure in the manufacturing industry will consistently be a nagging occupational health concern. Stakeholders initiating investigations, consequently leading to exposure abatement, are adjudged to have ideal hazard and risk perception. However the number of the complaints available in the reviewed literature seems minimal compared to the multitude of workplaces and occupational health stressors as well as occupational disease statistics, worldwide. Conclusively, there needs to be an improvement in the hazard and risk perception and appraisal of occupational hazards amongst different stakeholder at work if safe and healthy workplaces are to be attained.

That employees continue to be exposed to exposure levels above exposure limits amidst technological advancements in manufacturing processes highlights the need for renewed calls of making institution of occupational hygiene programs mandatory for all workplaces. This review paper also highlighted the continuing importance of the contemporary need and importance of occupational hygiene exposure measurements as a basis for informing risk and hazard perception amongst concerned stakeholders.

Further studies incorporating activities performed by labour inspectorates to those discussed in this current study will shed more light into the subject matter highlighted hereunder.

## Data Availability

Data supporting reported results can be found at https://www2a.cdc.gov/hhe/search.asp (accessed on 7 May 2021).
